# The chromosome‐level genome provides insight into the molecular mechanism underlying the tortuous‐branch phenotype of *Prunus mume*

**DOI:** 10.1111/nph.17894

**Published:** 2021-12-17

**Authors:** Tangchun Zheng, Ping Li, Xiaokang Zhuo, Weichao Liu, Like Qiu, Lulu Li, Cunquan Yuan, Lidan Sun, Zhiyong Zhang, Jia Wang, Tangren Cheng, Qixiang Zhang

**Affiliations:** ^1^ Beijing Key Laboratory of Ornamental Plants, Germplasm Innovation & Molecular Breeding National Engineering Research Centre for Floriculture Beijing Laboratory of Urban and Rural Ecological Environment Engineering Research Center of the Landscape Environment of the Ministry of Education Key Laboratory of Genetics and Breeding of Forest Trees and Ornamental Plants of the Ministry of Education School of Landscape Architecture Beijing Forestry University Beijing 100083 China

**Keywords:** genome assembly, molecular mechanism, plant architecture, *Prunus mume*, tortuous branch

## Abstract

- Plant with naturally twisted branches is referred to as a tortuous‐branch plant, which have extremely high ornamental value due to their zigzag shape and the natural twisting of their branches. *Prunus mume* is an important woody ornamental plant. However, the molecular mechanism underlying this unique trait in *Prunus* genus is unknown.
- Here, we present a chromosome‐level genome assembly of the cultivated *P. mume* var. tortuosa created using Oxford Nanopore combined with Hi‐C scaffolding, which resulted in a 237.8 Mb genome assembly being anchored onto eight pseudochromosomes.
- Molecular dating indicated that *P. mume* is the most recently differentiated species in *Prunus*. Genes associated with cell division, development and plant hormones play essential roles in the formation of tortuous branch trait. A putative regulatory pathway for the tortuous branch trait was constructed based on gene expression levels. Furthermore, after transferring candidate *PmCYCD* genes into *Arabidopsis thaliana*, we found that seedlings overexpressing these genes exhibited curled rosette leaves.
- Our results provide insights into the evolutionary history of recently differentiated species in *Prunus* genus, the molecular basis of stem morphology, and the molecular mechanism underlying the tortuous branch trait and highlight the utility of multi‐omics in deciphering the properties of *P. mume* plant architecture.

## Abstract 2

See also the Commentary on this article by Liu *et al.*, **235**: 8–10.

## Introduction

*Prunus* is a genus of shrubs or trees in the Rosaceae family and contains *c*. 30 species, which are mainly distributed in the northern temperate zone. *Prunus* subfamilies have high economic and ornamental value and have an important position in gardens worldwide, as *Prunus* plants have colourful and attractive flowers, leaves and fruits. To date, draft genome sequences have been completed for a total of eight *Prunus* species: *Prunus mume* (Zhang *et al*., 2012), *Prunus avium* (Wang *et al*., 2020), *Prunus persica* (Verde *et al*., 2013), *Prunus domestica* (Callahan *et al*., 2021), *Prunus dulcis* (Alioto *et al*., 2020), *Prunus armeniaca* (Jiang *et al*., 2019), *Prunus salicina* (Liu *et al*., 2020), and *Prunus yedoensis* (Baek *et al*., 2018). *Prunus mume* (which is also called mei), which has varying flower types, colourful corollas, a pleasant fragrance and an attractive plant architecture, originated in the Yangtze River Basin in southern China and expanded throughout East Asia 4000–5000 yr ago. As an early spring woody ornamental plant native to China, mei has long been a unique symbol of Chinese culture (Zhang *et al*., 2012, 2018). In 2012, Zhang *et al*. assembled a reference genome of *P. mume* using a highly wild plant and Illumina GA II technology, which was the first genome among *Prunus* subfamilies of the Rosaceae family. The estimated genome size was 280 Mb, and 84.6% (237 Mb) of its genome was assembled, with a contig N50 of 31.8 kb (Zhang *et al*., 2012). Based on genome data, *P. mume* accessions and three other *Prunus* species were resequenced to investigate the genetic architecture of floral traits and plant domestication history (Zhang *et al*., 2018). The publication of the *P. mume* genome represented a milestone for the genetic analysis of important ornamental traits of *Prunus* species.

To adapt to complex environments and compete for light and nutrients, plants have developed complex and diverse plant architectures. Plant architecture is extremely important to the growth, productivity and landscaping of crop plants, fruit trees, and flowering plants. Woody ornamental plants can be classified as straight‐branch, pendulous‐branch or tortuous‐branch types. A plant with naturally twisted branches is referred to as a tortuous‐branch plant. Tortuous branches exhibit an overall upward growth trend, and stem sections are naturally tortuous in a zigzag pattern, resulting in a peculiar but graceful shape (Zheng *et al*., 2018). After thousands of years of cultivation, > 300 varieties of *P. mume* with unique ornamental traits have been developed in China and Japan (Zhang *et al*., 2018). Among them, *P. mume* var. tortuosa is the only tortuous branch variety and has particularly high ornamental value because of its naturally tortuous branches and attractive flowers, which play important roles in urban landscaping. Naturally tortuous‐branch traits have been discovered in a few plant species, such as *Poncirus trifoliata* L. Raft var. monstruosa (Martínez‐Alcántara *et al*., 2013), *Salix matsudana* ‘Tortuosa’ (Lin *et al*., 2007), *Corylus avellana* L. ‘Montebello’ (Mehlenbacher & Smith, 2002), and *Morus alba*. var. tortuosa (Chen, 1981; Chen & Zhang, 1990), and in some zigzag‐shaped tea trees (Cao *et al*., 2020). The phenotype of tortuous‐branch traits is complex and is determined by several factors, such as branching angle, internode length and branch type. The results of previous studies have shown that the tortuous‐branch trait might be related to secondary growth, cytoskeleton, hormone regulation, geotropic growth, and environmental factors (Zheng *et al*., 2018). To date, several candidate genes associated with tortuous‐branch traits have been identified. Mutations resulting in tortuous branches have been found to be closely related to hormone regulatory genes. Grape plants with a mutation in the *GAI* gene and *GAI‐like* mutants of *Morus alba* are insensitive to gibberellic acid (Boss & Thomas, 2002; Sopian, *et al*., 2009). The *Arabidopsis* mutants *axr1* and *lop1*, which are closely related to auxin regulation, exhibit curved inflorescences (Lincoln *et al*., 1990; Carland & McHale, 1996). *Arabidopsis sgr2/4* mutants and transgenic plants expressing *AtCYCD3* also showed curled leaves and curved inflorescences (Fukaki *et al*., 1996; Yamauchi *et al*., 1997; Fujihira *et al*., 2000; Kato *et al*., 2002). In woody plants, transgenic plants expressing the *PtrHB2/7* and *PtoCYCD3;3* genes exhibit a tortuous‐branch phenotype (Robischon *et al*., 2011; Zhu *et al*., 2013; Guan *et al*., 2021), but the molecular mechanism of these tortuous branch traits remains unclear.

Owing to the limitations of second‐generation sequencing technology, there are several missing sequences and fragments in the *P. mume* genome that affect single‐nucleotide polymorphism (SNP) marker screening and the prediction of major candidate genes during genome‐wide association study (GWAS) and quantitative trait locus (QTL) mapping analyses (Zhang *et al*., 2018). Third‐generation sequencing technology (PacBio and Nanopore) can compensate for missing genomic regions that are difficult to assemble due to sequencing errors, repeat regions, heterochromatin, genomic polymorphisms and second‐generation sequencing preferences (Zheng *et al*., 2021b). Here, using Oxford Nanopore technology (ONT) combined with Hi‐C scaffolding, we constructed a chromosome‐level genome for *P. mume* var. tortuosa and analysed its evolutionary and genomic signatures. New evidence of controlling the formation of tortuous branches was found by combined analyses of transcriptomic data. This study provides the most comprehensive *Prunus* genome to date and a theoretical basis for understanding the regulatory mechanisms of plant architecture in woody ornamental plants.

## Materials and Methods

### Plant materials

Fresh young leaves used for genome sequencing were collected from *P. mume* var. tortuosa plants grown in a glasshouse at Beijing Forestry University, China. Regarding stem morphology, a very small number of branches that develop during the growing season of *P. mume* var. tortuosa grow straight; the tortuous‐branch phenotype is lost, and the original type (straight branch) is restored. These branches serve as rare experimental control materials. Because the development speed of the two different branches (tortuous vs straight) is different, it is impossible to obtain materials with exactly the same development period. However, the development speed of stem tip is basically the same, so we selected leaf buds and stem tips for further transcriptome sequencing. The data for the branching phenotype were collected from our previous study (Zhang *et al*., 2018). *Arabidopsis thaliana* (Col‐0) plants were grown in pots containing a mixture of turf peat, vermiculite, and sand (3 : 1 : 1, v/v) in a growth chamber with 60–75% relative humidity and an average temperature of 22 ± 2°C. Cool‐white fluorescent bulbs provided a photosynthetic photon flux density of 200 µmol m^−2^ s^−1^.

### Genome sequencing and assembly

High‐quality genomic DNA fragments of *P. mume* var. tortuosa were extracted from fresh young leaves using the cetyl‐trimethylammonium bromide (CTAB) method (Murray & Thompson, 1980). Then, the concentration and integrity of genomic DNA were detected via a qubit fluorometer and agarose gel electrophoresis. Two strategies were used to sequence the genome in our study. First, short‐read libraries were constructed using the BGI‐seq 500 platform. The raw data were subsequently filtered using SOAPnuke software (Chen *et al*., 2018) (filtering parameters: ‐t 10,0,12,0 ‐M 2 ‐l 10 ‐q 0.1 ‐n 0.05 ‐Q 2 ‐G), after which nucleotide sequence database (NT) alignment was employed to estimate the clean data sample quality using Blast software (Altschul *et al*., 1990). Then, *K*‐mer analysis was performed on the *P. mume* var. tortuosa genome by GenomeScope (Vurture *et al*., 2017) to preliminarily determine the genome size, heterozygous conditions and repetitive sequence information. The high‐throughput sequencing data were preliminarily assembled using SOAPdenovo (Li *et al*., 2010). Next, high‐quality genomic DNA fragments were used to construct long‐read libraries on the Nanopore platform. The raw data were assembled twice using the SMARTdenovo tool (Schmidt *et al*., 2017). Afterwards, the assembled contigs were polished by Pilon using short‐read sequence data (Walker *et al*., 2014).

### Hi‐C analysis and pseudochromosome construction

Fresh young leaves collected from *P. mume* var. tortuosa were crosslinked using formaldehyde at a concentration of 1%, and complexes containing biotin‐labelled compounds were constructed using a restriction enzyme (*Hin*d III). Illumina sequencing libraries were constructed using the biotinylated Hi‐C ligation products (Belton *et al*., 2012). The raw paired‐end reads were subsequently filtered by Hi‐C‐Pro (Servant *et al*., 2015) and aligned with the initial assembly reads. Based on the theory of intrachromosomal interactions, the scaffolds were sorted and assembled onto chromosomes using Juicer (Durand *et al*., 2016) and 3D *de novo* assembly (3D‐DNA) (Dudchenko *et al*., 2017). Benchmarking sets of universal single‐copy orthologues (Busco) software was used to assess the integrity of the genome assembly (Simão *et al*., 2015). We sampled hundreds of genomes and considered single‐copy orthologous genes that occurred in > 90% of the genomes as orthologous gene groups, after which we compared the homologous genes in the genome assembly results to assess the integrity of the genome assembly.

### Genome annotation

The *P. mume* var. tortuosa genome was annotated using genomic sequences, as well as repeated sequences, gene structure information, gene function information and noncoding RNAs. Repeated sequences were annotated according to the homologue method by RepeatMasker (VanBuren *et al*., 2018; Xu *et al*., 2018) using the Repbase database (Bao *et al*., 2015). RepeatModeler (Flynn *et al*., 2020), Piler (Edgar & Myers, 2005), RepeatScout (Price *et al*., 2005), Trf (Benson, 1999) and Ltr‐Finder (Xu & Wang, 2007) were used to annotate repeated sequences *de novo*. Three methods were used to annotate gene structures. First, Augustus (Stanke *et al*., 2006), GlimmerHMM (Majoros *et al*., 2004) and GenScan (Burge & Karlin, 1997) were used for *de novo* predictions according to the *P. mume* var. tortuosa genome. Second, the protein sequences of seven related species were selected for homologous annotation using GeneWise (Madeira *et al*., 2019). Next, transcript annotations were performed according to the RNA‐sequencing (RNA‐Seq) results using Hisat (Kim *et al*., 2015), StringTie (Kovaka *et al*., 2019), Trinity (Grabherr *et al*., 2011), Pasa (Haas *et al*., 2003) and TransDecoder (Onimaru *et al*., 2018). Each selected annotation satisfied at least one *de novo* prediction, with a short coding DNA sequence (CDS) length (≤ 150 bp) and transposable element (TE) overlap ratio of < 0.2. Gene functions were annotated via protein databases, including the SwissProt/TrEMBL (Bairoch & Apweiler, 2000), Kyoto Encyclopedia of Genes and Genomes (KEGG) (Ogata *et al*., 1999), InterPro (Mitchell *et al*., 2019) and Gene Ontology (GO) (Ashburner *et al*., 2000) databases, using protein sequences whose structures had been annotated. The annotations of noncoding RNAs included ribosomal RNAs (rRNAs), transfer RNAs (tRNAs), small nuclear RNAs (snRNAs) and microRNAs (miRNAs), as described previously (Lowe & Eddy, 1997; Griffiths‐Jones *et al*., 2005).

### Comparative genomic and genome evolutionary analyses

Orthologous groups were obtained from *P. mume* var. tortuosa and 13 other angiosperms using OrthoFinder (Emms & Kelly, 2015). Orthogroup species overlap was investigated via correlation analysis using the corrplot package in R. The MCL inflation of default parameters (1.5) was used as the cluster granularity setting (Dongen, 2000), and alignment with FFT‐NS‐2 in Mafft was then performed (Katoh *et al*., 2005). Gene trees of all the orthologous groups and a species tree were constructed using FastTree (Price *et al*., 2009). To further determine the phylogenetic relationships among the species, single‐copy genes were selected, and sites with coverage of < 85% were removed from 14 species, after which a species tree was constructed with the JTT+G+I model for amino acid sequences and the GTR+G+I model for nucleotide sequences in RAxML v.8.2.4 (Stamatakis, 2014).

To estimate the divergence times of plant species, single‐copy genes were extracted. A phylogenetic tree was constructed with RAxML v.8.2.4 with the best amino acid substitution model – the JTT model (Stamatakis, 2014). Clade support was assessed using a bootstrapping algorithm with 1000 alignment replicates. The divergence times of plant species were calculated by MCMCtree included in Paml (v.4.7a, RRID: SCR_014932) (Yang, 2007) with the following parameters: ‐‐rootage 500 ‐clock 3 ‐alpha 0.431879. Two calibration points were selected from the TimeTree website (http://www.timetree.org) as normal priors to reduce age, referencing speciation times of 98–117 million yr ago (Ma) for the divergence between *A. thaliana* and *Malus domestica* and 46–74 Ma for that between *M. domestica* and *Rosa chinensis*. Expansion and contraction of gene families were identified according to the divergence predicted by the phylogenetic tree. Syntenic blocks and paralogous and orthologous gene pairs were identified using MCScanX (Wang *et al*., 2012). ParaAT software was used to convert amino acid sequences to nucleotide sequences. Then, the synonymous substitutions per synonymous site (Ks) values were calculated using the kaks_calculator package (Wang *et al*., 2010).

### Histochemical and histological analyses

About 0.5 cm long stem segments of 1‐yr‐old branch of *P. mume* var. tortuosa were fixed in formaldehyde–acetic acid solution (formaldehyde : glacial acetic acid : ethanol (1 : 1 : 18)) for 24 h, dehydrated in a graded ethanol series, and embedded into paraplasts. The samples were then sectioned to a thickness of 8 μm using a Leica RM2235 rotary microtome. The sections were subsequently stained with safranin and fast green and then screened by a panoramic scanner (3DHistech, Budapest, Hungary).

### RNA extraction and transcriptome analysis

Total RNA was extracted from the different samples using an RNAprep Pure Plant Kit (Tiangen, Beijing, China). Then, the quality of the total RNA was measured using a NanoDrop 2000 spectrophotometer (Thermo Scientific, Wilmington, DE, USA). High‐quality total RNA was selected and used to construct complementary DNA (cDNA) libraries according to the manufacturer’s instructions. The cDNA libraries were evaluated for quality and sequenced on an Illumina HiSeq platform, and paired‐end reads were generated. Clean reads were obtained by removing adaptor sequences and low‐quality sequence reads from raw reads and aligned to the reference genome sequence using TopHat2 software (Kim *et al*., 2013).

The expression levels of genes were quantified based on the position information of mapped reads using Cufflinks software (Trapnell *et al*., 2012) and estimated via the fragments per kilobase of transcript per million fragments (FPKM) (Florea *et al*., 2013). To further examine the biological replicates between samples, principal component analysis (PCA) and correlation analysis of all possible pairs of samples were performed according to the expression levels of genes using R software. Differential expression analysis of each pair was performed using DESeq2 (parameters: false discovery rate (FDR) < 0.05 and |log_2_(fold change) (FC)| > 1) (Pertea *et al*., 2015). Differentially expressed genes (DEGs) were clustered using the pheatmap package of R and annotated using the GO and KEGG databases (Ogata *et al*., 1999; Ashburner *et al*., 2000). Protein–protein interactions were predicted according to orthologous genes using the Search Tool for the Retrieval of Interacting Genes/Proteins (STRING) database (Szklarczyk *et al*., 2015).

### Gene cloning and plant transformation

To verify the biological function of candidate genes, the CDSs of four *PmCYCD* genes were obtained via PCR using specific primers (Supporting Information Table S1) and inserted into the plant expression vector pBI121, which harbours a kanamycin resistance gene (Zheng *et al*., 2021a). The vector constructs were subsequently transformed into *Agrobacterium tumefaciens* (*GV3101*), which were integrated into the *A. thaliana* genome via the floral‐dip method (Clough & Bent, 1998). We ultimately obtained > 10 positive plants for each gene after we screened the seeds on solid Murashige & Skoog (MS) media supplemented with 50 mg l^−1^ kanamycin and cross‐detected the transgenic *Arabidopsis* plants at the DNA and messenger RNA (mRNA) levels.

### Morphological microscopy comparisons

Leaf epidermal cells of young leaves from wild‐type (WT) and transgenic *Arabidopsis* were removed with tweezers. The epidermal cells were placed on a slide in a drop of water, covered with a coverslip, and observed and imaged under a Zeiss light microscope (Docuval; Carl Zeiss, Germany).

### RNA extraction and qRT‐PCR analysis

Total RNA was extracted from 30‐d‐old transgenic *Arabidopsis* plants using a MiniBEST Plant RNA Extraction Kit (TaKaRa, Dalian, China) and analysed by using a NanoDrop 2000c spectrophotometer (Thermo Scientific). Total RNA was reverse transcribed into cDNA, and quantitative reverse transcription polymerase chain reaction (qRT‐PCR) detection was performed with SYBR Premix EX Taq II (TaKaRa, Shiga, Japan) on a CFX96 Real‐Time PCR Detection System (Bio‐Rad, Hercules, CA, USA). All the primers used for qRT‐PCR are shown in Table S1. The expression level of each sample was normalized to that of the *Atactin* reference gene and determined using the 2‐delta‐delta cycle threshold (*C*_t_) method (Livak & Schmittgen, 2001).

## Results

### Genome sequencing and assembly of *P. mume* var. tortuosa

A diploid (2*n* = 2*x* = 16) *P. mume* var. tortuosa plant was used for whole‐genome sequencing and chromosome‐level assembly via short‐read sequencing (BGI‐seq 500) and long‐read sequencing (Oxford Nanopore), respectively. After filtering out low‐quality reads with SOAPnuke software, a total of 44.66 Gb (168×) of clean reads from the BGI‐seq 500 platform and 11.7 Gb (50×) of Oxford Nanopore long reads were obtained (Tables S2, S3). Furthermore, we carried out NT database comparative evaluation by BLAST, and our results showed that the top six species in the comparison were *P. mume*, *P. persica*, *P. avium*, *P. tomentosa*, *P. armeniaca* and *P. yedoensis*, which were closely related to the target species, indicating that there was no obvious exogenous pollution (Table S4). Based on *K*‐mer (*K* = 17) distribution analysis, *P. mume* var. tortuosa was estimated to have a genome size of 261.6 Mb, with a high heterozygosity of 0.75% and a high repetitive sequence content of 52.12% (Fig. S1; Table S5). All the contigs of the Oxford Nanopore long reads were extended using SMARTdenovo to generate an assembly with a total contig length of 237.7 Mb (91% of the genome), consisting of 225 contigs with a contig N50 size of 2.75 Mb, and the largest contig size was 12.6 Mb (Tables S3, S6).

Hi‐C scaffolding generated a total of 120.9 million read pairs, with an average mapping ratio of 84.5% (Fig. S2). After mapping the Hi‐C reads against the assembly of *P. mume* var. tortuosa, 26.6 million valid pairs were used for the Hi‐C analysis (Table S7). Juicer (v.1.5) and 3D‐DNA software were applied to construct chromosomal‐level scaffolds. Of the 234.9 Mb of scaffold sequences, 98.78% of the bases were anchored to eight pseudochromosomes with lengths ranging from 20.3 Mb to 47.0 Mb (Tables S8, S9; Fig. S3). The final chromosome‐level genome assembly of *P. mume* var. tortuosa was 237.8 Mb, with a scaffold N50 of 29.4 Mb, and the largest scaffold size was 47.0 Mb (Table 1).

**Table 1:** Major indicators of the *Prunus mume* var. tortuosa, *P. mume*, and *P. persica* genomes.

Parameter	*Prunus mume* var. tortuosa	*Prunus mume* (wild‐type)	*Prunus persica* cv. Chinese Cling
Sequencing platform	BGI‐seq 500, BioNano, Hi‐C	Illumina GA II	Illumina, PacBio, Hi‐C
Estimate of genome size	262 Mb	280 Mb	249.8 Mb
Repetitive sequence	52.12 (%)	45 (%)	46.36 (%)
Heterozygosity	0.75 (%)	0.03 (%)	0.28%
Total number of contigs	225	45 811	300
Total length of contigs	237.7 Mb	219.9 Mb	247.33 Mb
N50 of contigs	2.75 Mb	31.8 kb	4.13 Mb
N90 of contigs	546.9 kb	5.77 kb	—
Maximum length of contigs	12.6 Mb	—	—
Minimum length of contigs	45.8 kb	—	—
GC content	37.46 (%)	—	37.59%
Average length of contigs	1.06 Mb	—	—
Total number of scaffolds	32	29 989	135
Total length of scaffolds	237.8 Mb	237.1 Mb	247.34 Mb
N50 of scaffolds	29.4 Mb	577.8 kb	29.68 Mb
N90 of scaffolds	24.0 Mb	86.0 kb	—
Maximum length of scaffolds	47.0 Mb	2.87 Mb	—
Minimum length of scaffolds	5000 bp	—	—
Complete Buscos	96.4 (%)	—	96.4%
Number of protein‐coding genes	29 706	31 390	26 335
Average length of transcripts	3897	2514	2919.19
Average length of coding sequences	1258	1146	1312.83
Number of annotated genes	26 015	25 905	25 625
Number of microRNAs	97	209	242
Number of transfer RNAs	546	508	564
Number of ribosomal RNAs	274	125	298
Number of small nuclear RNAs	1004	287	552

**Fig. 1:**
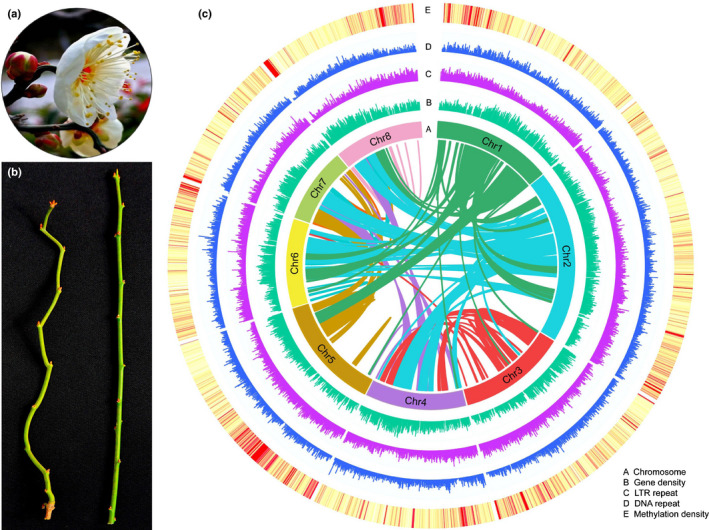
Synteny and distribution of genomic and epigenomic features of the *Prunus mume* var. tortuosa genome. (a) Flowers of *P. mume* var. tortuosa used in this study. (b) Tortuous branches (left) and straight branches (right) of *P. mume* var. tortuosa. (c) Genomic and epigenomic features of *P. mume* var. tortuosa. The intermediate circles from the outer circle to the inner circle (A–E) represent chromosomes, gene densities, long terminal repeats (LTRs), DNA repeats and methylation densities. The coloured lines in the centre of the circle represent synteny relationships among gene blocks.

The complete genome was estimated to have a 37.46% GC content, a 98.85% mapping rate of short reads, and 96.4% complete Buscos (Fig. 1; Tables S10, S11). These results indicated that the *P. mume* var. tortuosa genome assembly was highly consistent and complete.

### Genome annotation

To obtain a reliable gene structure, we used RNA‐Seq data from various tissues to facilitate accurate genome annotation. A total of 112.47 Mb (47.29%) of the *P. mume* var. tortuosa genome was composed of repetitive sequences, and 45.43% of these repeat sequences were *de novo* sequences (Table S12). A total of 46.19% of the genome sequences were annotated as TEs, of which long terminal repeats (LTRs) were predominant, accounting for 29.30% of the whole genome. Moreover, through a combination of repeat modellers and LTR_FINDER, *de novo* TEs were speculated to account for 45.14% of the genome (Table S13). We used *de novo*, homology‐based, and transcriptomic methods for gene structure prediction. The gene structure characteristics of *P. mume* var. tortuosa conformed to those of most species, but the number of introns with a length of *c*. 100 bp was distinctly less than that in *Arabidopsis*. Based on the embryophyta_odb9 database, 95.4% of the single‐copy genes were fully annotated by Busco analysis (Table S14).

A total of 29 706 protein‐coding genes were predicted in the *P. mume* var. tortuosa genome (Table 1). Specifically, 26 015 of the 29 706 proteins (87.57%) were annotated by using the SwissProt, KEGG, TrEMBL, and InterPro databases (Fig. S4; Table S15). Compared with the number of genes annotated in the previous *P. mume* genome (31 390 protein‐coding genes), the number of protein‐coding genes in the *P. mume* var. tortuosa genomes was reduced by 1683, but the average length of transcripts and CDSs was greater in the latter. A total of 1921 noncoding RNAs (Table S16) – 97 miRNAs, 546 tRNAs, 274 rRNAs, and 1004 snRNAs – were annotated, accounting for 0.1% of the genome. The complete chloroplast genome size of *P. mume* var. tortuosa was 157 903 bp and the genome exhibited a quadripartite structure (Fig. S5). A portion of the mitochondrial genome (39 578 bp) was identified and annotated, including the *rps1/4*, *nap4/7* and *atp1* genes (Fig. S6).

### Comparative genomic and genome evolutionary analyses

**Fig. 2:**
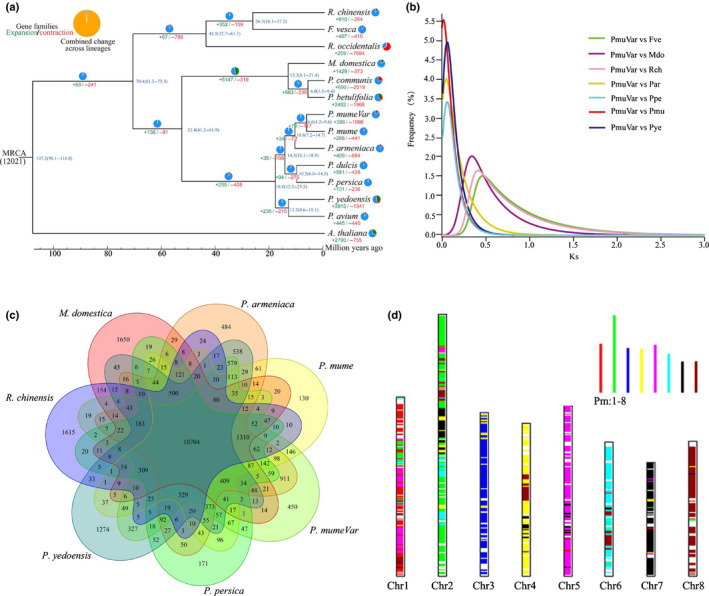
Evolution of the genome and gene families. (a) Phylogenetic tree with single‐copy orthologues from 14 species for determining divergence times. The expansion or contraction of gene families is shown via pie charts. (b) Ks distribution for orthologues between *Prunus mume* var. tortuosa and seven species (*Fragaria vesca*, *Malus domestica*, *Rosa chinensis*, *P. persica*, *P. yedoensis*, *P. armeniaca* and *P. mume*). (c) The shared and unique gene families were compared among seven closely related Rosaceae species (*M. domestica*, *R. chinensis*, *P. persica*, *P. yedoensis*, *P. armeniaca*, *P. mume* and *P. mume* var. tortuosa). Each number represents the number of gene families. (d) Chromosome‐level collinearity patterns between *P. mume* var. tortuosa and *P. mume*. Syntenic *P. mume* chromosomal regions are overlaid on the *P. mume* var. tortuosa chromosomes.

We assigned 461 952 (92.4%) genes to 35 512 orthogroups using OrthoFinder. Fifty per cent of them were in orthogroups with 18 or more genes (the G50 of which was 18) and were assigned to the largest 7621 orthogroups (the O50 of which was 7621) (Fig. S7). There were 7976 orthogroups with genes from all species present, 291 of which were entirely of single‐copy genes. However, 10 704 shared genes were present across the seven species, most likely representing the core genes of Rosaceae, and 450 genes were specific to the *P. mume* var. tortuosa genome (Fig. 2c). Moreover, 23 542 collinear gene pairs were identified between *P. mume* var. tortuosa and *P. mume*, accounting for 79.2% and 75.0% of the total number of genes in these genomes, respectively (Fig. S8). These differences might be attributed to the expansion/contraction of gene families during the evolution of these species (Table S17). Compared with that of *P. mume*, the number of gene families of *P. mume* var. tortuosa that expanded/contracted was prominent – 1482 gene families (Fig. 2a). In contrast, the numbers of gene families of *P. mume* that expanded and contracted was significantly lower – 226 and 441, respectively (Fig. 2a). Moreover, a total of 23 934 gene trees were constructed from the orthogroups using RAxML. A species tree comprising 1167 orthogroups was constructed, with a minimum of 92.9% of the species having single‐copy genes in any orthogroup. Moreover, 4441 orthogroups supported the best root from the observed 4443 well‐supported, nonterminal duplications, and *A. thaliana* was selected as the best outgroup for the species tree (Figs 2a, S7). To investigate the phylogenetic position of *Prunus*, single‐copy genes were selected from 13 Rosaceae genomes spanning major lineages of *Fragaria* and *Rosa*. Based on different data types and tree inference methods, our results showed that the speciation times were *c*. 41.2–61.9 Ma due to the divergence between *Prunus* and *Pyrus*, *P. mume* was more closely related to *P. armeniaca* than to the other species as the result of recent differentiation, and the ancestor of the two species split *c*. 10.8 Ma (Fig. 2a). These results suggested that *P. mume* was the most recently differentiated species in *Prunus*.

The *Vitis vinifera* genome has not undergone recent genome duplication, and this ancestral arrangement is common in many dicotyledonous plant species. Therefore, comparing chromosomal segments of plant genomes to those of *V. vinifera* is a powerful approach for describing gene and chromosomal duplication events (Jaillon *et al*., 2007; Verde *et al*., 2013). Comparisons of the *P. mume* var. tortuosa genome structure with the *V. vinifera* genome structure showed that the chromosomal arrangement changed significantly after the speciation of *P. mume* var. tortuosa (Fig. S9). A total of 662 syntenic genomic blocks, which included 18 318 collinear gene pairs, were identified between *P. mume* var. tortuosa and *V. vinifera* (Fig. S9), suggesting that there was a triplicate arrangement (an ancestral γ event), which has been confirmed in the *P*. *mume* genome (Zhang *et al*., 2012). Synonymous substitutions were characterized at synonymous nucleotide sites (Ks) between collinear homoeologues within or between *P. mume* var. tortuosa and seven other species of the Rosaceae (Figs 2b, S10). We calculated the Ks values of orthologues between *P. mume* var. tortuosa and seven other species with different Ks peaks (Fig. 2b), and the results of which indicated divergent evolutionary rates among these eight species. In addition, based on previous studies of synonymous substitutions per site per year (Lynch & Conery, 2000; Blanc & Wolfe, 2004), we calculated the estimated times of the Ks peaks to have occurred at *c*. 50 and 108.7 Ma, respectively, suggesting the absence of a recent whole‐genome duplication (WGD) event. Consistent with this argument, duplicate blocks of *P. mume* var. tortuosa were located only in regions with blocks of the same hexaploid ancestor (Fig. 1b).

### Chimaerism and characteristics of *P. mume* var. tortuosa

Tortuous branches are naturally one of the main ornamental characteristics of *P. mume* var. tortuosa. However, we newly revealed genotypes with tortuous and straight branch types (Fig. 1a,b), providing valuable control materials for subsequent omics research.

**Fig. 3:**
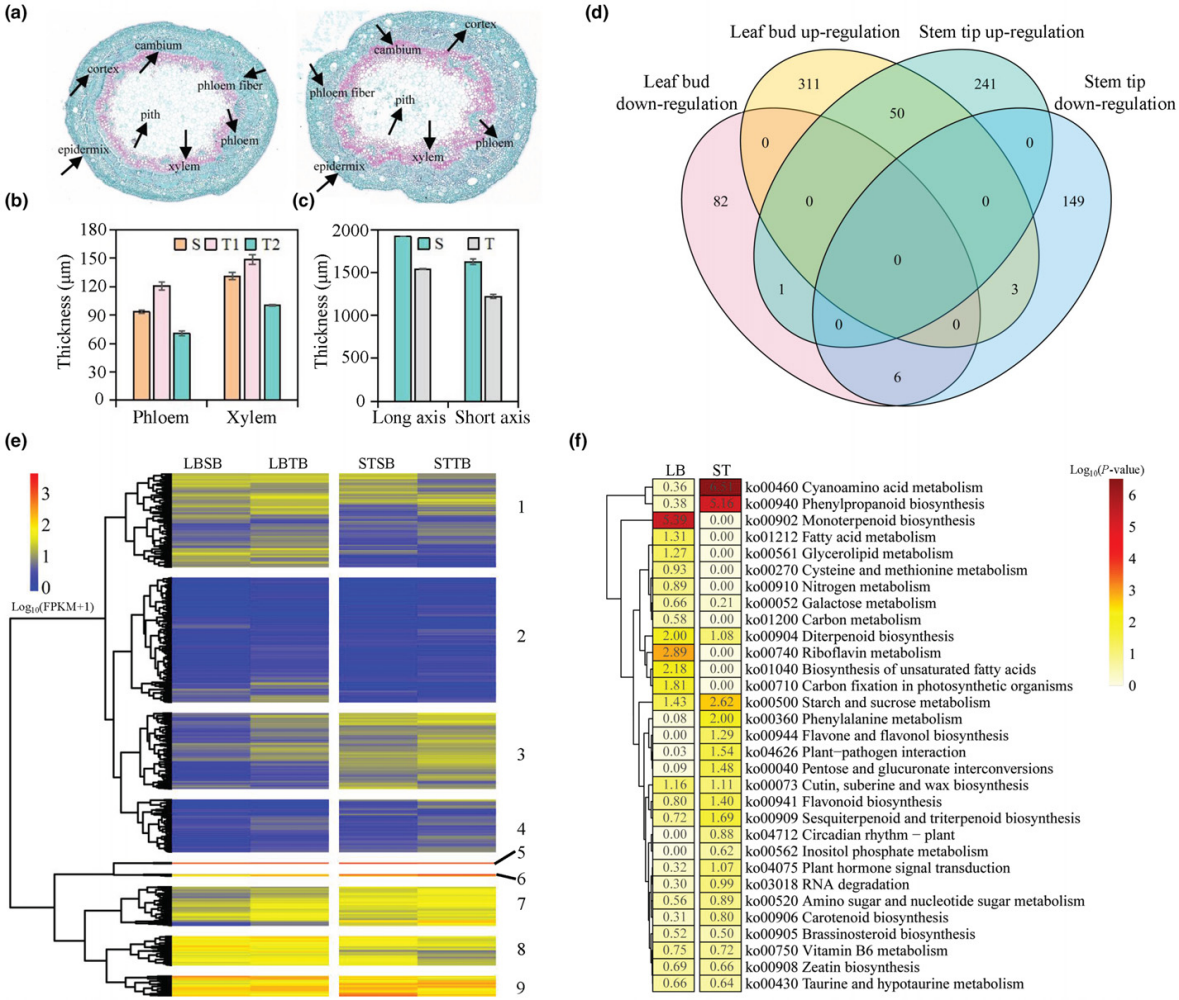
Anatomical characteristics and transcriptomes of straight and tortuous branches of *Prunus mume* var. tortuosa. (a) Paraffin sections and saffron‐solid green tissue staining were used to observe cross‐sections of straight and tortuous branches. (b) Thickness of phloem and xylem in straight and tortuous stems. (c) Thickness of straight and tortuous branches along the long axis and short axis. S, straight stem; T, tortuous stem; T1, tortuous stem on the bending side; T2, tortuous stem on the opposite side. The error bars represent ± SD. (d) Venn diagram showing the shared and unique genes among the differentially expressed genes (DEGs), including those in leaf bud and stem tip samples. (e) Heatmap of the log_10_(fold change) of all the DEGs. The rows and columns represent the genes and samples, respectively, clustered by similarity within the gene expression profile. (f) Heatmaps of −log_10_ enrichment *P*‐values for the 10 most‐enriched KEGG pathways among the DEGs. LB represents the KEGG enrichment results of DEGs between the leaf buds of straight and tortuous branches. ST represents the KEGG enrichment results of DEGs between the stem tips of straight and tortuous branches.

To reveal the differences between straight and tortuous branches, their histological structures were analysed via phloroglucinol–hydrochloric acid and saffron‐solid green tissue staining. The bends of lignified branches were mainly concentrated in the phloem, while the bends of nonlignified branches were mainly concentrated in the leaf buds (Fig. S11). Compared with straight stems, tortuous stems had fewer phloem fibres, and their growth was not symmetric along the vertical axis (Fig. 3a–c). Both the phloem and the xylem on the bent side were thicker than those in the straight stems, but both the phloem and the xylem on the other side were thinner than those in the straight stems (Fig. 3b). Cross‐sections of the straight stems were oval, but those of the tortuous stems were irregularly oval with two concave regions, and the tortuous stems were thinner than the straight stems were on both the long axis and the short axis (Fig. 3c). These results suggested that the changes in the leaf buds might be causing the asymmetric xylem and phloem development. Tortuous branch formation is therefore caused by asymmetric xylem and phloem development and occurs early in the development of *P. mume* var. tortuosa stems.

### Transcriptome divergence between straight and tortuous branches

To evaluate the effects of gene transcription levels on stem morphology, we compared the gene expression levels between straight and tortuous branches, including for two groups of materials: leaf buds and stem tips. Cluster analysis of gene expression profiles showed that leaf buds and stem tips were clustered into two major classes instead of straight branches and tortuous branches (Fig. S12), which indicated that the expression level of genes related to growth and development was the main factor. At the same time, the straight branch and tortuous branch samples could be divided into two groups in each major class (Fig. S12), which indicated that the genes were differentially expressed in the early stage of stem morphogenesis.

We ultimately identified 453 and 450 DEGs in the leaf bud (straight vs tortuous) and stem tip (straight vs tortuous) samples, respectively, of which 60 were differentially expressed in both groups (Fig. 3d). We found that 83.3% of the shared DEGs were upregulated and that 10.0% of the shared DEGs were downregulated in these two periods. Hierarchical clustering was used to reconstitute all the DEG clusters between the straight and tortuous branch samples. The majority of the 843 stem morphogenesis‐related genes exhibited tissue‐specific expression divergence, including genes in clusters 1, 3, 4, 6, 7 and 8 (Fig. 3e). In total, 245 and 233 DEGs were assigned GO terms for the DEGs in the leaf bud (straight vs tortuous) and stem tip (straight vs tortuous) samples, respectively (Tables S18, S19). In the biological process category, metabolic process, single‐organism process, and cellular process were the most highly represented groups in both periods. Within the cellular component category, DEGs that corresponded to cells and cell parts were the most abundant. Among the significantly enriched biological processes, many DEGs involved in lignin biosynthetic processes (GO: 009809) and adenosine triphosphate (ATP) catabolic processes (GO: 0006200) showed obvious differences in both the leaf buds and stem tips. KEGG pathway annotation of these genes revealed high‐level functions and biological processes, including cyanoamino acid metabolism (ko00460), monoterpenoid biosynthesis (ko00902), plant hormone signal transduction (ko04075) and brassinosteroid (BR) biosynthesis (ko00905) (Fig. 3f; Tables S20, S21).

### Weighted gene coexpression network analysis (WGCNA) of tortuous branch‐related transcriptome characteristics

**Fig. 4:**
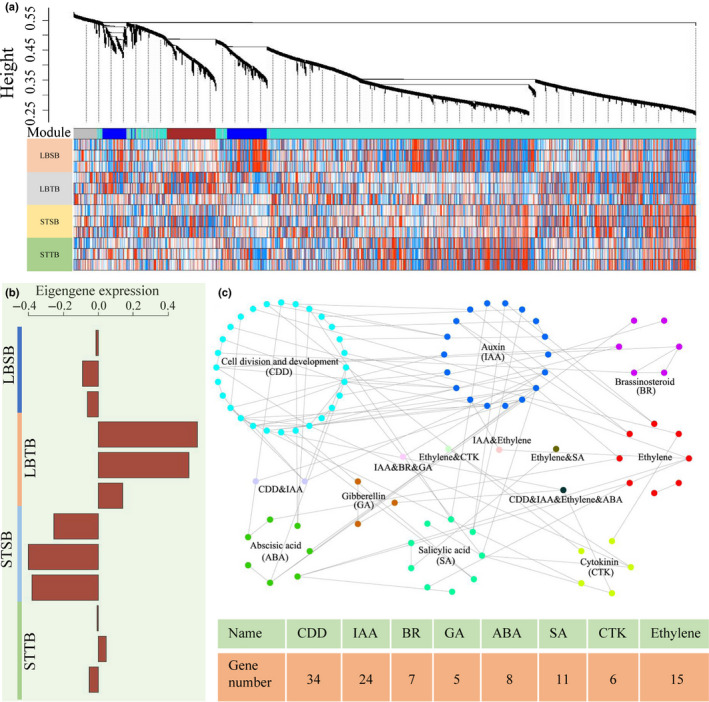
Establishment of a coexpression network. (a) Gene dendrogram and corresponding module colours. The clustering was based on the expression levels of 17 756 genes. (b) Relationships between the brown modules and expression of corresponding eigengenes across samples in the brown modules. (c) Network component analysis of proteins associated with cell division, development and plant hormones within the brown modules.

The power of the coexpression networks could provide deep insight into the complex molecular mechanisms underlying the differences between straight and tortuous branches. First, the samples and genes were filtered according to their gene expression profiles. We removed genes and samples with an absence rate greater than or equal to 10%. A total of 17 756 genes from 12 samples were ultimately clustered into three modules (brown, blue and turquoise modules) using weighted gene coexpression network analysis (WGCNA) (Fig. 4). We focused on the brown module, which was significantly associated with both straight and tortuous branch traits (Fig. 4b). The genes in the brown module (1233 genes) were upregulated overall in both the leaf buds and the shoot tips of the tortuous branch samples. A total of 96 of these genes were closely related to the regulation of cell division, development and plant hormones, and their expressed proteins closely interacted with each other (Fig. 4c). Moreover, one of these genes was potentially involved in multiple biological regulatory processes (Fig. 4c). Specifically, AMP1 might play a role in balancing and restricting the meristem‐promoting activity of auxin signalling and might be involved in ethylene and gibberellin signalling pathways or in a parallel pathway that controls cell and hypocotyl elongation and cellular organization (Vidaurre *et al*., 2007; Huang *et al*., 2015). In addition, *PmCYCD* genes are differentially expressed in the straight and tortuous branches of *P. mume* and respond to multiple plant hormone treatments (Zheng *et al*., 2019). Taken together, the results indicated that genes associated with cell division, development and plant hormones play an important role in the formation of tortuous branch traits.

### Regulatory pathway for tortuous branch traits

**Fig. 5:**
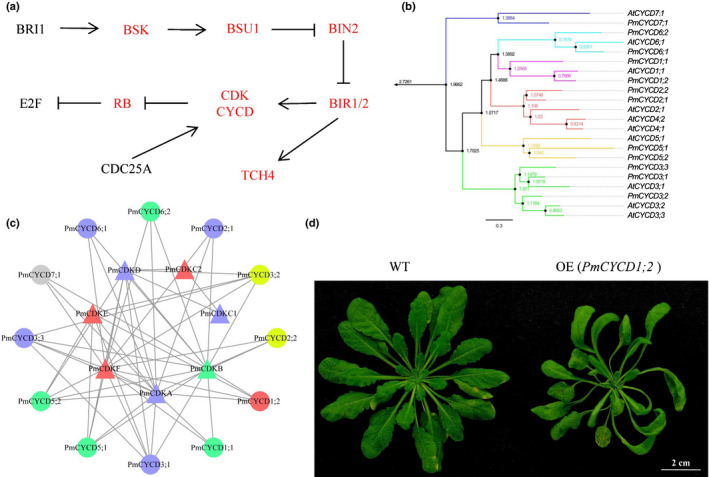
Functional verification of key genes that regulate stem development. (a) Reported pathways and genes that might be involved in the regulation of stem morphology. The red‐labelled gene had collinear support and was retained in *Prunus mume* var. tortuosa. (b) Phylogenetic tree of *CYCD* genes in seven subfamilies in *P. mume* var. tortuosa and *Arabidopsis thaliana*. (c) Interaction networks of PmCYCDs and PmCDKs based on the sequences of *A. thaliana* orthologues in the STRING database. The red, grey, and blue colours represent upregulated, nonexpressed, and downregulated genes, respectively, in the leaf buds and stem tip samples of tortuous branches compared with straight branches. The green colour represents upregulated genes in the leaf buds and downregulated genes in the stem tips. The yellow colour represents downregulated genes in the leaf buds but upregulated genes in the stem tips. (d) Phenotype of *PmCYCD1;2* OE plants compared with wild‐type (WT) plants.

To speculate about the molecular regulatory mechanism of stem morphology, we identified the high‐confidence DEGs between straight and tortuous branches, and some members had very strong correlations with plant cell division and development and were usually involved in plant hormone signal transduction and cellular senescence pathways. We speculated that the molecular pathway might be involved in the regulation of tortuous‐branch traits based on known interactions and gene coexpression, as shown in Fig. 5(a). A total of 27 orthogroups, which included 46 orthologous genes, were identified according to the homologous sequences in *A. thaliana*, and 37% of the orthogroups contained genes with more than one copy in *P. mume* var. tortuosa (Table S22). First, brassinosteroid insensitive 1 (BRI1) is a receptor with dual specificity kinase activity in response to BR binding. Probable serine/threonine‐protein kinase (BSK) acts as a positive regulator of BR signalling downstream of the receptor kinase BRI1 and positively regulates serine/threonine‐protein phosphatase (BSU1) (Tang *et al*., 2008). Moreover, BSU1 inactivates the negative regulator of BR signalling BIN2 by dephosphorylation (Kim *et al*., 2011). Brassinosteroid‐resistant 1/2 (BZR1/2) positively regulates the expression of xyloglucan endotransglucosylase/hydrolase protein (TCH4) and D‐type cyclin protein (CYCD) genes (He *et al*., 2005). CYCD proteins in turn interact with cyclin‐dependent kinases (CDKs), which are activated by cell division cycle (CDC) proteins. These complexes act as transcriptional repressors of retinoblastoma (RB)‐related protein target genes and further affect the regulation of E2F transcription factors (E2Fs) (Boudolf *et al*., 2004).

CDKs regulate cell division, which is crucial for plant growth, development and morphogenesis. By combining orthogroup information and domain characteristics, we identified seven *CDK* genes among 1114 genes containing a phinase domain (PF00069) from the *P. mume* var. tortuosa genome (Fig. S13). Gene duplication event analysis showed that the duplication of *PmCDKC1* (PmuVar_Chr2_5573) and *PmCDKC2* (PmuVar_Chr1_0803) might be the result of *P. mume* var. tortuosa segmental duplications (Fig. S14). Only the *PmCDKB* gene (PmuVar_Chr1_1946) of *P. mume* var. tortuosa was found in the orthogroup containing *AtCDKB1‐1/2*, while only the *CDKB* gene (*Pm022893*) of *P. mume* was found in the orthogroup containing *AtCDKB2‐1/2* (Fig. S15). We found that the expression level of *PmCDK* genes and the degree of difference were different between the leaf buds and stem tips, which indicated a potential specific expression pattern of *PmCDK* during *P. mume* var. tortuosa development (Fig. S16). Half of the *PmCDK* family members were upregulated in the tortuous branches. The expression of *PmCDKC1* was upregulated in the tortuous branches, but the *PmCDKC2* gene produced by its replication was downregulated (Fig. S16). Moreover, the *PmCDKB* gene was upregulated in the leaf buds but downregulated in the stem tips, suggesting that the expression of the *PmCDKB* gene might be tissue or time specific. The gene expression patterns further suggested a primary role for the expression and regulation of *PmCDKs* in stem morphological development.

Cyclins, which constitute a prominent class of cell division regulators, play an extremely important role in plant growth and development. A total of 46 cyclin genes were identified and further divided into 10 subfamilies according to their orthologues in *A. thaliana* (Fig. S17). Genome synteny analysis showed that 52.5% of genes were duplicated in *P. mume* var. tortuosa, which involved 10 pairs of collinearity events and four pairs of tandem events (Table S23). Moreover, 71.1% of the genes had significant genomic synteny with grape genes (Table S24), including all the members of the *CYCD* subfamily (12 *CYCD* genes) (Fig. 5b). Seven *PmCYCD* genes might be products of *P. mume* var. tortuosa segmental duplications, as these genes were present in two syntenic gene blocks on the chromosomes of *P. mume* var. tortuosa, which corresponded to homologous genes on the chromosomes of grape. Notably, *PmCYCD3;1*, *PmCYCD3;2* and *PmCYCD3;3* had syntenic relationships with only one *CYCD* gene in grape. Compared with those of straight branches, five *PmCYCD* genes in the leaf buds of tortuous branches were upregulated, and six genes were downregulated (Fig. 5c). The expression of the *PmCYCD1;2* gene was upregulated in both the leaf buds and the stem tips, while its duplicate gene *PmCYCD1;1* was upregulated only in the leaf buds. The expression levels of *PmCYCD2;2*, *PmCYCD3;1*, *PmCYCD3;2* and *PmCYCD6;1* were two‐ to five‐fold different between the leaf buds and stem tips, respectively, indicating that these genes might have specific expression patterns. To verify the functions of D‐type cyclins, four *PmCYCDs* (*PmCYCD1;1*, *PmCYCD1;2*, *PmCYCD2;1* and *PmCYCD3;1*) were cloned and transformed into *A. thaliana*. Compared with control *Arabidopsis* plants (Fig. S18), *PmCYCD1;2* overexpression (OE) seedlings showed curled rosette leaves (Fig. 5d). The number of epidermal cells of the OE (*35S::PmCYCD1;2*) plants was less than half of that of the WT, and number of cells was twice as large (Fig. S19). The qRT‐PCR showed that 11 vascular cambium development‐ and division‐related genes were upregulated in the OE plants (Fig. S20). The *AtCDKB1;1*, *AtCDKB1;2*, *AtKNAT1* and *AtAS2* genes were upregulated more than three times in OE plants compared with the WT plants. Taken together, these results suggested that the specific expression of *PmCYCDs* might lead to abnormal development of the vascular cambium through cell division, which might affect the morphogenesis of plant architecture.

## Discussion

Due to the rapid development of genome sequencing technology worldwide, higher‐quality genomes are urgently needed. A long‐term strategic genomic research plan that is not limited to WT materials should be formulated in consideration of important cultivated species with important ornamental value and economic value. Using a combination of Oxford Nanopore technology, Illumina short reads and Hi‐C scaffolding, we successfully assembled a high‐quality genome for *P. mume* var. tortuosa, and 98.78% of the sequences were anchored onto eight pseudochromosomes. Compared with a previous draft genome from wild *P. mume* with a lower contig N50 value (31.77 kb) and scaffold N50 value (577.82 kb), the newly assembled genome was greatly improved, with a contig N50 of 2.75 Mb and a scaffold N50 of 29.4 Mb (Table 1). Moreover, the high‐level genome prominently increased the average length of transcripts and decreased the number of gene segments and pseudogenes. Although cultivated varieties are highly heterozygous, the quality index of the *P. mume* var. tortuosa genome was still higher than that of the other genomes of other *Prunus* species (Table S25). Thus, this reference genome could be useful for studies concerning molecular breeding, genetics and evolution in *P. mume* and other *Prunus* species, especially the genetic mechanism underlying woody plant architecture traits.

The *Prunus* genus, which belongs to the Rosaceae family, includes > 30 species of flowering trees and shrubs that are mostly deciduous plants. Most members are diploid (2*n* = 2*x* = 16), although a few polyploids are included. Several sequenced genomes in the genus are relatively small, *c*. 2–3 times the size of the *Arabidopsis* genome (Jung *et al*., 2019). Since cross‐pollination is very common in the *Prunus* genus, reproduction, successful pollination, zygote formation and seed development are essential. Phylogenetic analysis indicated that diversification of the *Prunus* genomes occurred during 41.2–61.9 Ma, followed by the successive split of the *Prunus* genus during 12.5–23.5 Ma. This result is supported by the divergence of the *Prunus* genome from the Rosaceae family during the Palaeocene and the continuous disintegration of the *Prunus* genus during the Eocene (Baek *et al*., 2018). In addition, our phylogenomic analyses supports *P. mume* being the most recent species to separate from all other extant *Prunus* species. Compared with the other *Prunus* species, *P. mume* has evolved more attractive flowers, colourful corollas and varying flower types. The expansion or contraction of a specific category of genes related to early flowering, floral scent and colour may be involved in the diversification of *P. mume* flowering. Many new accessions have been developed by crossing *P. mume* with apricot or plum plants, suggesting that reproductive barriers between these *Prunus* species are likely not present (Zhang *et al*., 2018; Bao *et al*., 2019; Shi *et al*., 2020). The high diversity in floral traits may be due to the broad expression of homologues of floral genes during evolution; this high diversity could also result from interspecific hybridization and additional introgression by backcrossing between closely related flowering *Prunus* species.

*Prunus mume* var. tortuosa is the only variety with the tortuous branch trait, which makes it an ideal perennial plant for studying stem morphology. However, the molecular mechanism underlying this tortuous branch trait is unclear. To investigate the molecular mechanism of tortuous branch traits, the differentially expressed stem morphology‐related genes were screened by coupling microstructure information with RNA‐Seq results. As a result, we identified a putative molecular regulatory pathway of the tortuous branch trait. Phytohormones play vital roles in stem development and plant architecture formation. For example, both *Morus alba GAI*‐like mutants and grape tortuous‐branch mutants formed by *GAI* gene mutations are insensitive to GA3 (Boss & Thomas, 2002; Sopian *et al*., 2009). Two *Arabidopsis* auxin mutants, *axr1* and *lop1*, also exhibit curved inflorescences because of a disruption in auxin synthesis (Lincoln *et al*., 1990; Carland & McHale, 1996). The responsible genetic network mainly involves hormone signal transduction and cell senescence pathways, of which BR signal transduction has been elucidated in plants (Kim *et al*., 2009; Kim & Wang, 2010). Studies on cellular senescence pathways have focused on animals and humans (Muñoz‐Espín & Serrano, 2014; Childs *et al*., 2015), but the functions of many homologous genes have been demonstrated in plants (Magyar *et al*., 2005; Hirano *et al*., 2008, 2011; Yao *et al*., 2018). In terms of the genetic network, PmCDKs and PmCYCDs are homologues of CDKs and CYCDs in *Arabidopsis*, respectively, and both act as regulators of cell cycle progression (Riou‐Khamlichi *et al*., 1999, 2000). Most CYCDs form a stable complex with CDKs, and OE of some *CYCD*/*CDK* genes can promote the S phase transition, which indicates that the CDK–CYCD complex can regulate the G1‐to‐S phase transition (Masubelele *et al*., 2005; Menges *et al*., 2006; Hirano *et al*., 2008, 2011). *PmCYCDs* are involved in the response to exogenous hormone (naphthylacetic acid (NAA), 6‐benzylaminopurine (6‐BA), GA3, and abscisic acid (ABA)) and sucrose applications and regulate multiple processes involved in plant growth and development (Zheng *et al*., 2019). Asymmetric cell division is the foundation of multicellular organism development (Riou‐Khamlichi *et al*., 1999). OE of *PmCYCD1;2* in transgenic plants resulted in curled rosette leaves, similar to findings in *Arabidopsis* and tobacco (Riou‐Khamlichi *et al*., 2000; Yang *et al*., 2014; Linsmith *et al*., 2019). OE of *CYCD1;2* in *Populus tremula* × *Populus alba* resulted in decreased cell size and altered leaf morphology (Williams *et al*., 2015). Similarly, OE of *PtoCYCD3;3* promoted growth and caused leaf wrinkling and branching in transgenic poplar (Guan *et al*., 2021), indicating that *PmCYCDs* may lead to abnormal stem development, which may affect *P. mume* var. tortuosa plant architecture formation.

In summary, the regulation of tortuous branch traits is not caused by a single mechanism but instead results from the combined action of multiple mechanisms. Our current knowledge about the regulatory pathways involved in tortuous branch traits is still limited. *Prunus mume* var. tortuosa is currently the only woody plant with a unique plant architecture and whose genome has been sequenced; this genome sequence is therefore useful for studies concerning the mechanisms underlying the formation of important ornamental traits and for molecular breeding in *P. mume* and other *Prunus* species.

## Author contributions

QZ and TZ planned and designed the research. TZ, PL and XZ conducted the experiments and collected the materials. TZ, PL and XZ analysed the data. WL, LQ, LL, CY, LS, ZZ, JW and TC conducted the fieldwork and maintained the materials. PL, TZ, and XZ wrote the manuscript. TZ and QZ revised the manuscript, provided advice on the experiments and finalized the manuscript. All the authors have read and approved the final manuscript. TZ, PL and XZ contributed equally to this work.

## Supporting information


**Fig. S1** Analysis of genomic heterozygosity.
**Fig. S2** Statistics of comparisons between Hi‐C reads and the genome.
**Fig. S3** Heat maps representing chromosomal interactions.
**Fig. S4** Genome annotation.
**Fig. S5** Circular chloroplast genome of *Prunus mume* var. tortuosa.
**Fig. S6** Mitochondrial genome of *Prunus mume* var. tortuosa.
**Fig. S7** Orthogroups assigned to the whole genome according to OrthoFinder.
**Fig. S8** Comparison of the *Prunus mume* var. tortuosa genome with the *P. mume* genome.
**Fig. S9** Comparison of the *Prunus mume* var. tortuosa genome with the *Vitis vinifera* genome.
**Fig. S10** Ks distribution of paralogous genes among eight species.
**Fig. S11** Anatomical characteristics of straight and tortuous stems of *Prunus mume* var. tortuosa.
**Fig. S12** Leaf buds and stem tip gene expression correlations between straight branches and tortuous branches.
**Fig. S13** Phylogenetic tree of *CDK* genes.
**Fig. S14** Phylogenetic tree of *CDKC* genes from *Prunus mume* var. tortuosa and select plants.
**Fig. S15** Phylogenetic tree of *CDKB* genes from *Prunus mume* var. tortuosa and select plants.
**Fig. S16** Heat map showing the transcript abundance of *PmCDK* genes.
**Fig. S17** Phylogenetic tree of cyclins from *Prunus mume* var. tortuosa and *Arabidopsis thaliana*.
**Fig. S18** *PmCYCD* overexpression in *Arabidopsis thaliana*, resulting in a curled rosette leaf phenotype.
**Fig. S19** The cellular level phenotype of the *PmCYCD1;2* overexpression (OE) plants compared with wild‐type (WT) plant.
**Fig. S20** Relative expression levels of tortuous branch‐related genes.

**Table S1** Primer sequences used for quantitative reverse transcription polymerase chain reaction (qRT‐PCR) in this study.
**Table S2** Sequencing data of *Prunus mume* generated by the BGI‐seq 500 platform.
**Table S3** Statistical results of genome sequencing using the Oxford Nanopore platform.
**Table S4** Statistical results of NT comparisons among six species of *Prunus* plants.
**Table S5** Genome analysis results using the *K*‐mer method.
**Table S6** Genome assembly results based on Nanopore sequencing.
**Table S7** Statistical results of Hi‐C sequencing.
**Table S8** Assembly results of the genome based on the Hi‐C platform.
**Table S9** Statistical analysis of genome length and mounting rate at the chromosome level.
**Table S10** Statistical results of genome assembly evaluation according to Busco.
**Table S11** Statistical results of genome read depth/coverage assessment.
**Table S12** Summary of repeated sequences.
**Table S13** Transposable element (TE) statistical results for different repetition types.
**Table S14** Gene structure annotation and Busco evaluation results.
**Table S15** Gene functional annotation.
**Table S16** Noncoding RNA annotation results.
**Table S17** Genomic information concerning 12 species.
**Table S18** GO enrichment results of differentially expressed genes (DEGs) in the leaf buds between straight and tortuous branches.
**Table S19** GO enrichment results of differentially expressed genes (DEGs) in the stem tips between straight and tortuous branches.
**Table S20** KEGG enrichment results of differentially expressed genes (DEGs) in the leaf buds between straight and tortuous branches.
**Table S21** KEGG enrichment results of differentially expressed genes (DEGs) in the stem tips between straight and tortuous branches.
**Table S22** Genes related to putative regulatory pathways involved in stem morphology.
**Table S23** Duplicated *PmCYCD* gene pairs.
**Table S24** Synteny analysis of cyclin genes between *Prunus mume* var. tortuosa and *Vitis vinifera*.
**Table S25** Comparative analysis of the sequenced genomes of species in the *Prunus* genus.
Please note: Wiley Blackwell are not responsible for the content or functionality of any Supporting Information supplied by the authors. Any queries (other than missing material) should be directed to the *New Phytologist* Central Office.

## Data Availability

The whole‐genome sequences have been deposited in the National Centre for Biotechnology Information (NCBI) under BioProject ID PRJNA720973. The raw transcriptome RNA‐Seq data of tortuous and straight *P. mume* are available at the National Genomics Data Centre (NGDC) under accession no. PRJCA001153 (BioProject: CRA001273).
