# Supplementary material for: The chromosome‐level genome provides insight into the molecular mechanism underlying the tortuous‐branch phenotype of Prunus mume
Source: New Phytol. 2021 Dec 17;235(1):141–56. doi: 10.1111/nph.17894 (PMC9299681; doi:10.1111/nph.17894)
Supplement: Supplementary file 1 — Fig. S1 Analysis of genomic heterozygosity. Fig. S2 Statistics of comparisons between Hi‐C reads and the genome. Fig. S3 Heat maps representing chromosomal interactions. Fig. S4 Genome annotation. Fig. S5 Circular chloroplast genome of Prunus mume var. tortuosa. Fig. S6 Mitochondrial genome of Prunus mume var. tortuosa. Fig. S7 Orthogroups assigned to the whole genome according to OrthoFinder. Fig. S8 Comparison of the Prunus mume var. tortuosa genome with the P. mume genome. Fig. S9 Comparison of the Prunus mume var. tortuosa genome with the Vitis vinifera genome. Fig. S10 Ks distribution of paralogous genes among eight species. Fig. S11 Anatomical characteristics of straight and tortuous stems of Prunus mume var. tortuosa. Fig. S12 Leaf buds and stem tip gene expression correlations between straight branches and tortuous branches. Fig. S13 Phylogenetic tree of CDK genes. Fig. S14 Phylogenetic tree of CDKC genes from Prunus mume var. tortuosa and select plants. Fig. S15 Phylogenetic tree of CDKB genes from Prunus mume var. tortuosa and select plants. Fig. S16 Heat map showing the transcript abundance of PmCDK genes. Fig. S17 Phylogenetic tree of cyclins from Prunus mume var. tortuosa and Arabidopsis thaliana. Fig. S18 PmCYCD overexpression in Arabidopsis thaliana, resulting in a curled rosette leaf phenotype. Fig. S19 The cellular level phenotype of the PmCYCD1;2 overexpression (OE) plants compared with wild‐type (WT) plant. Fig. S20 Relative expression levels of tortuous branch‐related genes. [file NPH-235-141-s001.pdf]

New Phytologist Supporting Information

Article title: The chromosome-level genome provides insight into the molecular mechanism underlying the tortuous-branch phenotype of *Prunus mume*

Authors: Tangchun Zheng, Ping Li, Xiaokang Zhuo, Weichao Liu, Like Qiu, Lulu Li, Cunquan Yuan, Lidan Sun, Zhiyong Zhang, Jia Wang, Tangren Cheng and Qixiang Zhang

Article acceptance date: 20 November 2021

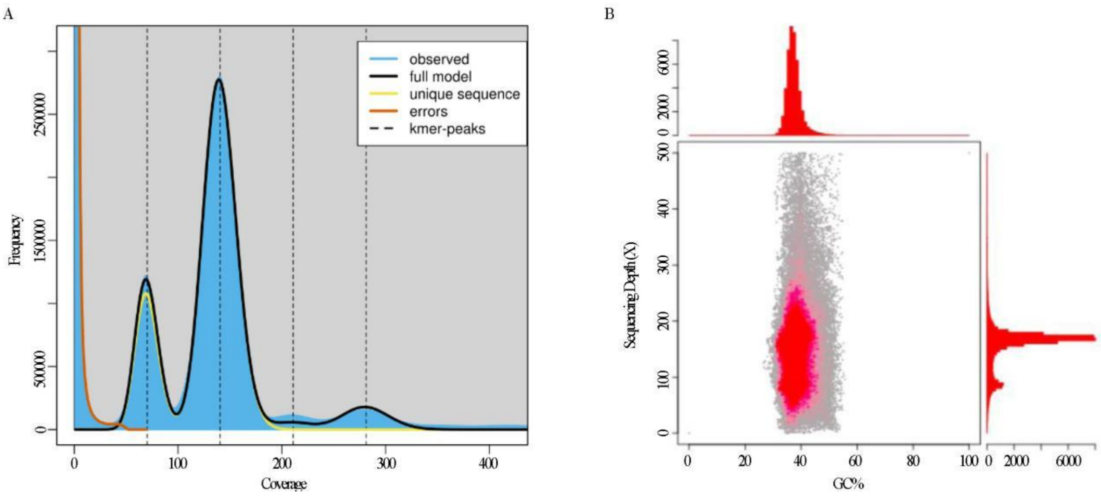

Fig. S1 Analysis of genomic heterozygosity. **A**. K-mer depth distribution curve. **B**. Distribution map of GC content and sequencing depth.

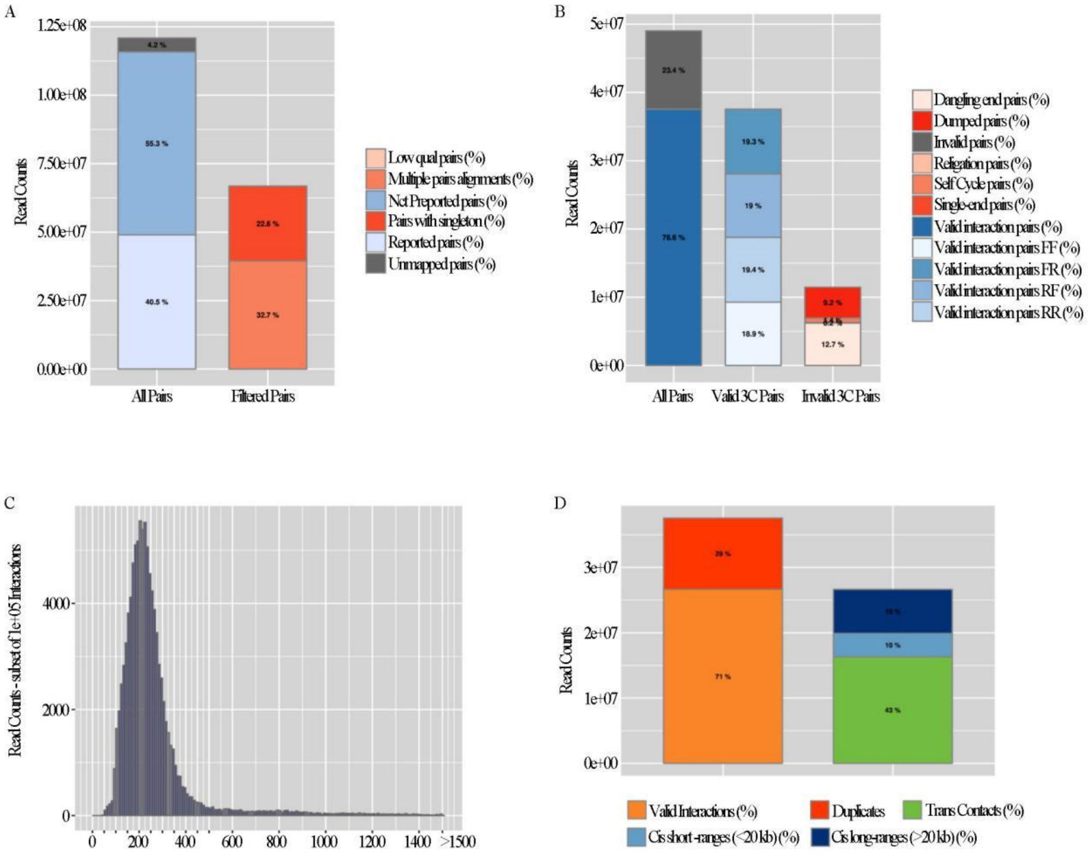

Fig. S2 Statistics of comparison between Hi-C reads and genome. **A**. Compare the proportion of positions, including all pairs and filtered pairs. **B**. The statistical results were compared with the reads of the enzyme digested fragment. **C**. The length distribution of reads on enzyme digested fragments. **D**. Percentage of repeat pairs of read pairs.

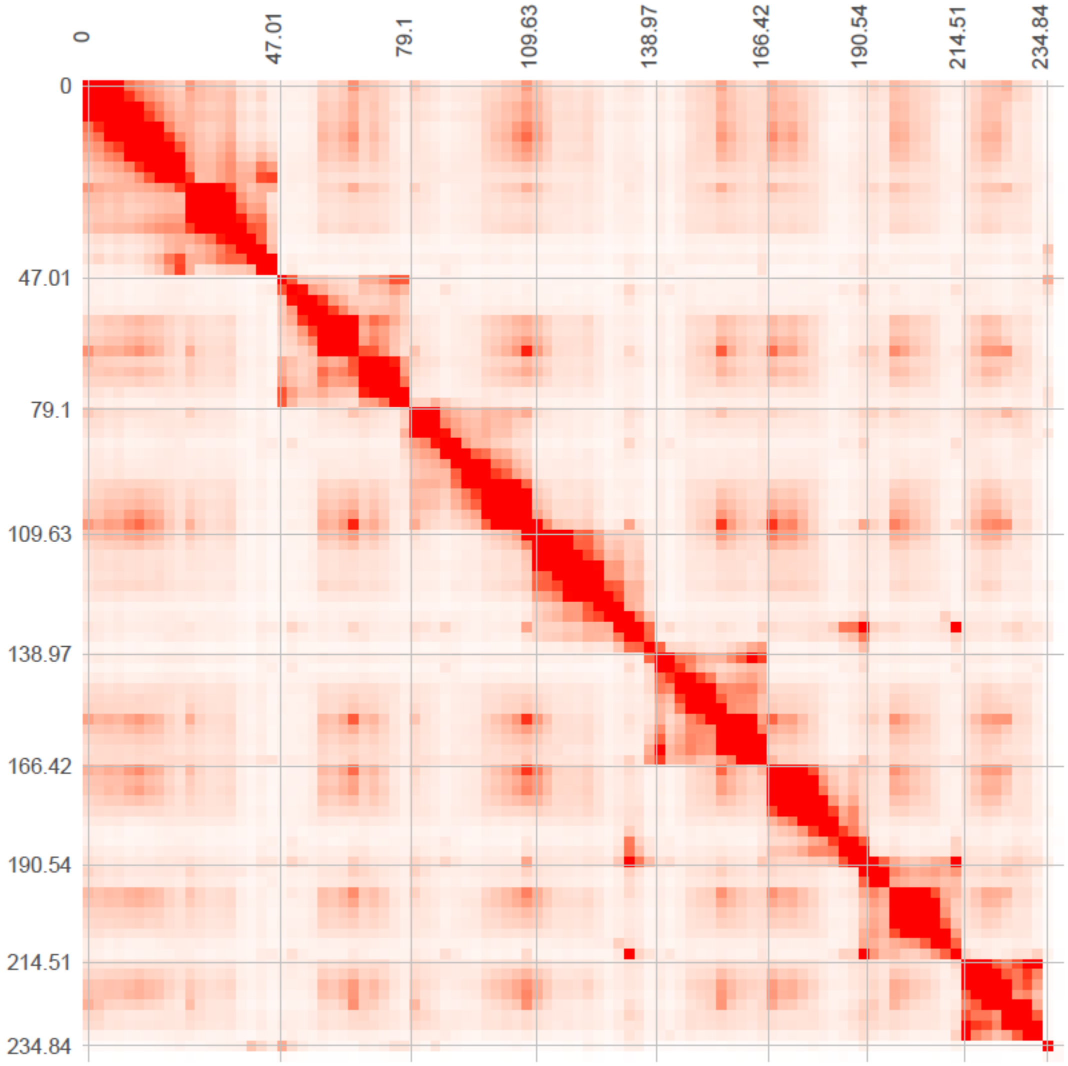

Fig. S3 Heat maps represent chromosomal interactions.

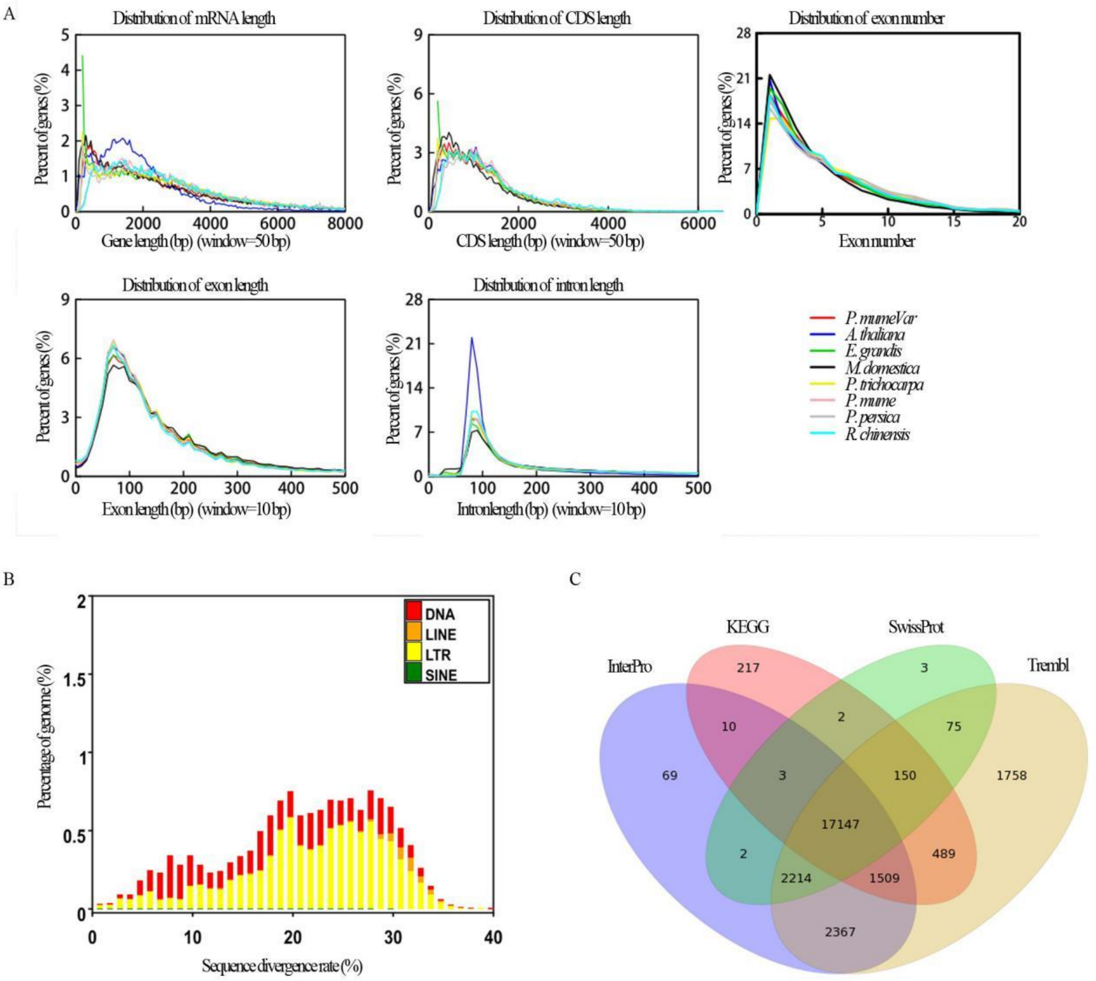

Fig. S4 Genome annotation. **A**. Comparison of genetic structural elements in related species. **B**. TE sequence distribution of different repetition types. **C**. Gene functional annotation results.

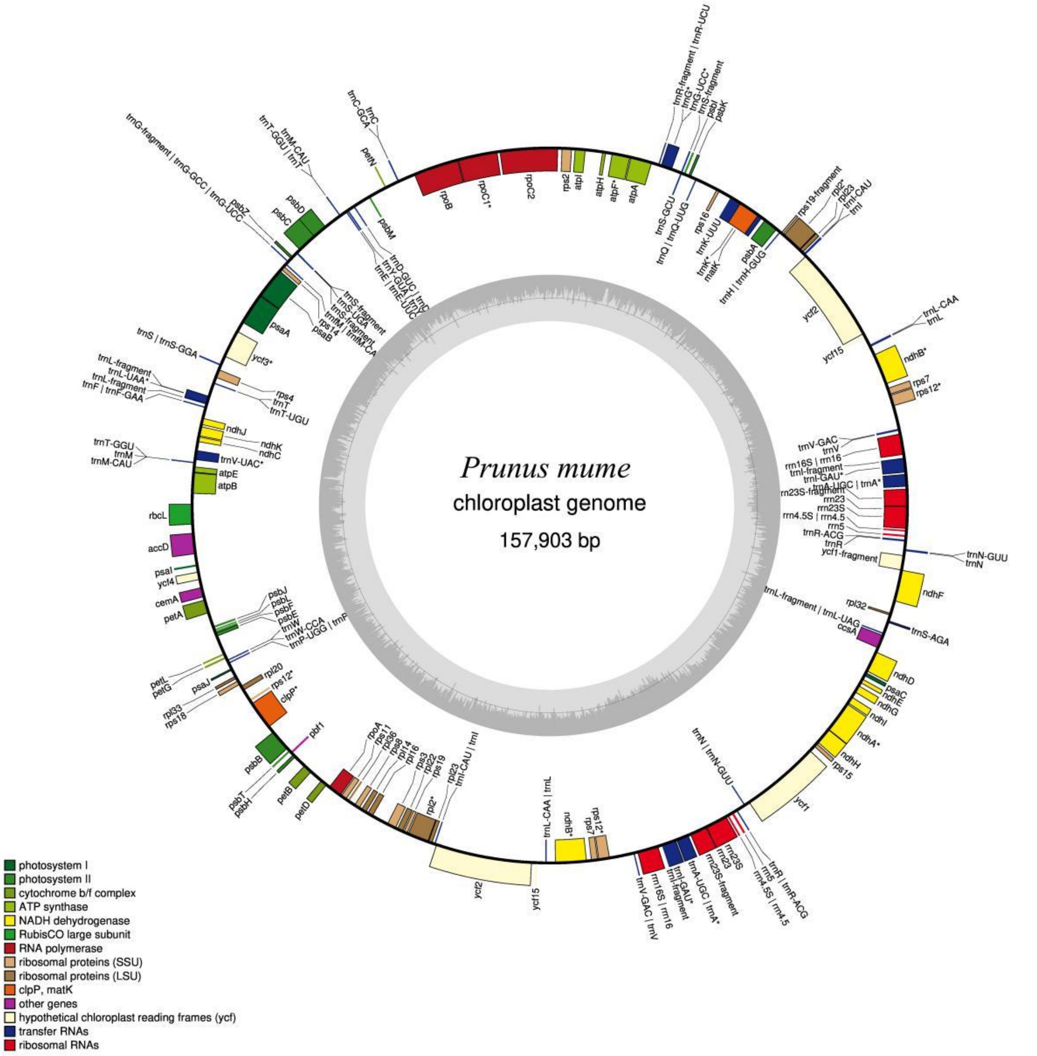

Fig. S5 The circular chloroplast genome of *P. mume* var. tortuosa. Colour-coded boxes indicated the genes in the genome. The figure was generated using Organellar Genome DRAW.

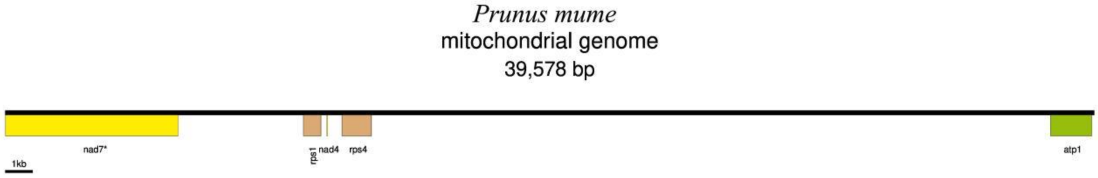

Fig. S6 The mitochondrial genome of *P. mume* var. tortuosa. Colour-coded boxes indicated the genes in the genome. The figure was generated using Organellar Genome DRAW.

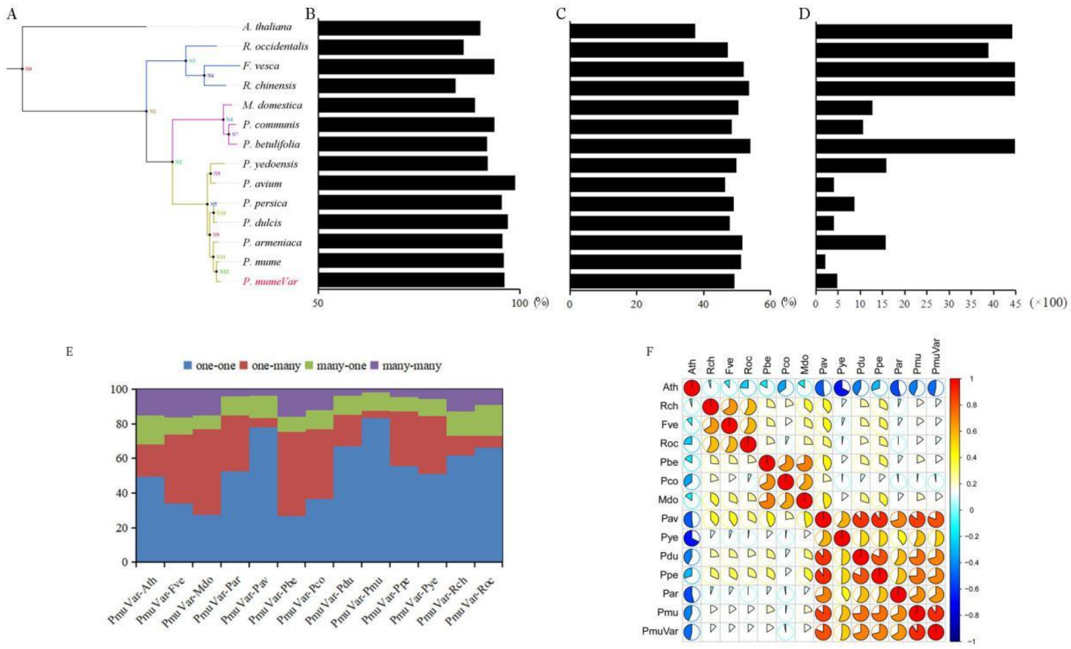

Fig. S7 The orthogroups were assigned in whole genome using OrthoFinder. **A**. A species tree was built using single-copy genes in any orthogroups and *A. thaliana* was selected best outgroup for species tree. **B**. Percentage of genes in orthogroups. **C**. Number of orthogroups containing species. **D**. Number of genes in species-specific orthogroups. **E**. Statistics of orthologues. **F**. Species overlaps were analyzed based on orthogroups.

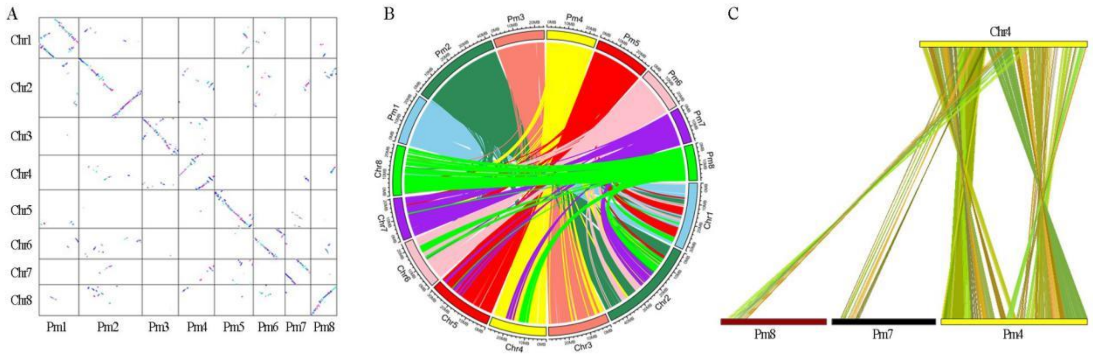

Fig. S8 Comparison of the *P. mume* var. tortuosa genome with the *P. mume*. **A**. Syntenic blocks between genomes. Dot plots of orthologs show chromosomal relationship between *P. mume* var. tortuosa and *P. mume*. **B**. Synteny patterns between genomic regions from *P. mume* var. tortuosa and *P. mume*. **C**. The genes of syntenic block are showed between chromosome 4 of *P. mume* var. tortuosa and the *P. mume* genome.

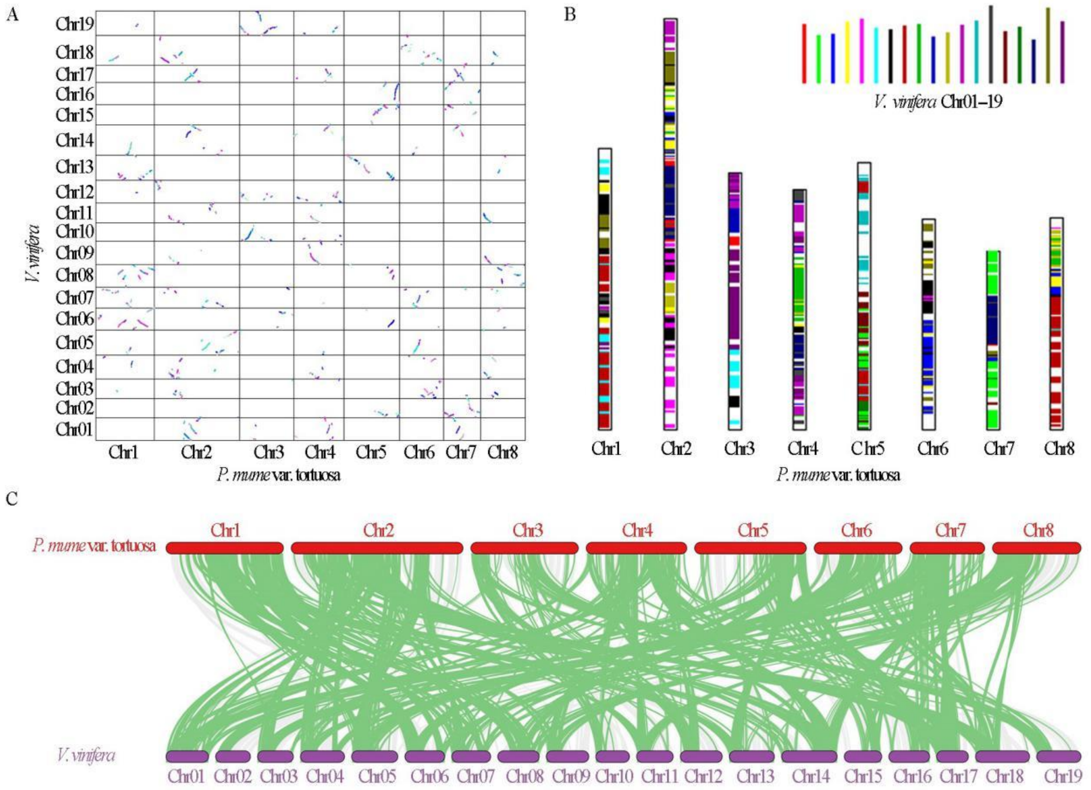

Fig. S9 Comparison of the *P. mume* var. tortuosa genome with the *V. vinifera*. **A**. Syntenic blocks between genomes. Dot plots of orthologs show chromosomal relationship between *P. mume* var. tortuosa and *V. vinifera*. **B**. Syntenic *V. vinifera* are painted onto *P. mume* var. tortuosa chromosomes. **C**. Synteny patterns between genomic regions from *P. mume* var. tortuosa and *V. vinifera*.

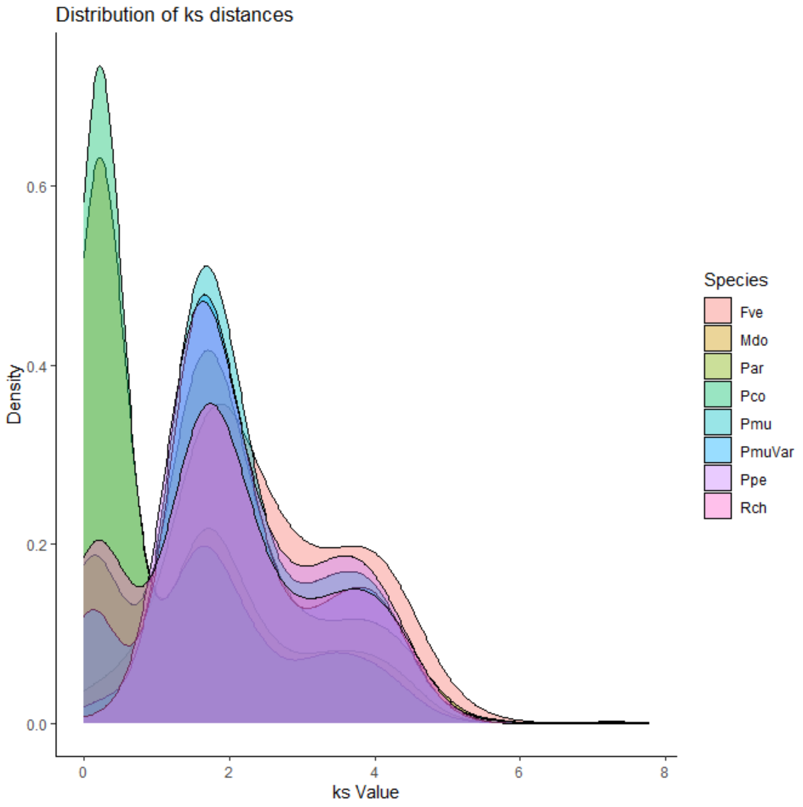

Fig. S10 The synonymous substitutions per synonymous site (ks) distributions of paralogous genes among 8 species. *F. vesca*, *M. domestica*, *R. chinensis*, *P. persica*, *P. yedoensis*, *P. armeniaca*, *P. mume* and *P. mume* var. tortuosa.

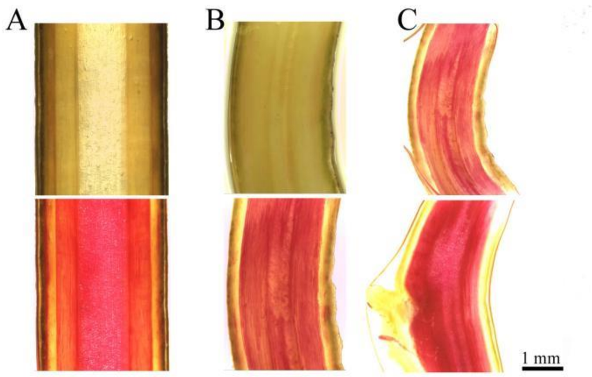

Fig. S11 Anatomical characteristics of straight and twisted stems in *Prunus mume* var. tortuosa. **A** and **B**. Bare-handed sections and phenol dyein were used to observe longitudinal section of straight and twisted branches. **C**. Bare-handed sections and phenol dyein were used to observe longitudinal section of a non-budding and budding stem.

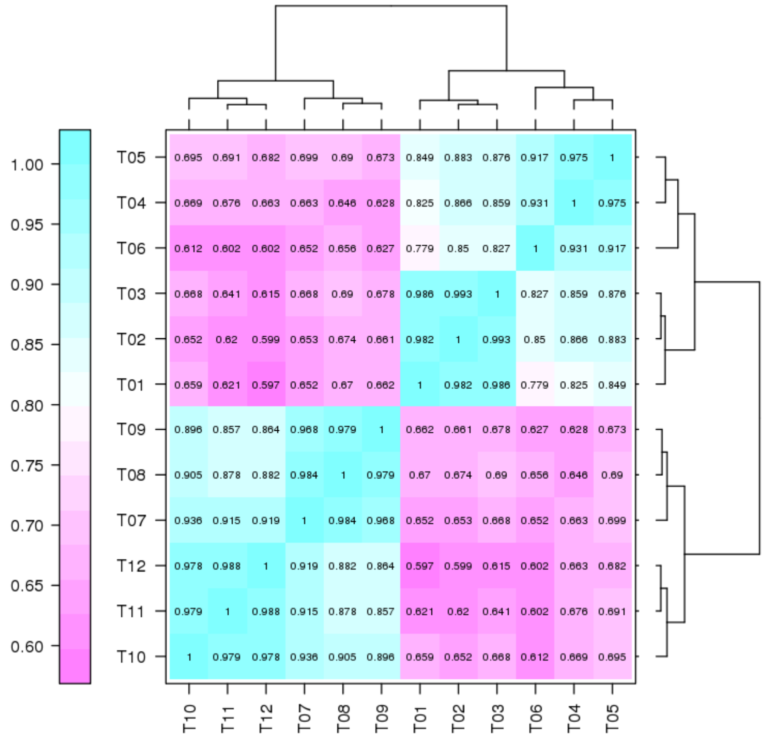

Fig. S12 The correlation of gene expression was analyzed between straight and twisted branches from the leaf bud and stem tip. T01 ~ 03 and T07 ~ 09 were three biological samples of leaf bud and stem tip in straight branches, respectively. T04 ~ 06 and T10 ~ 12 were three biological samples of leaf bud and stem tip in twisted branches, respectively. The scale was Pearson correlation coefficient and displayed in gradient color.

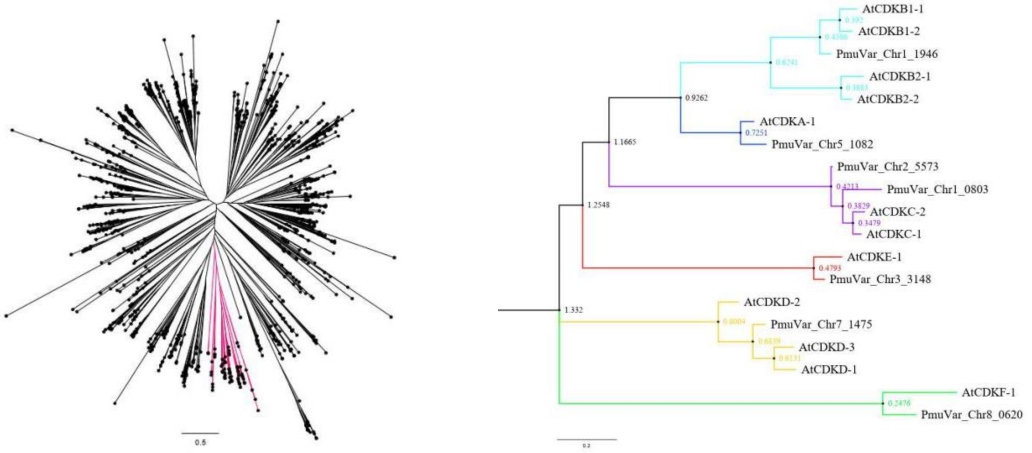

Fig. S13 The phylogenetic trees of *CDKs* genes. The phylogenetic tree on the left represents the Pkinase domain of the *P. mume* var. tortuosa and *AtCDK* genes. The branches of the tree marked in red contain the *AtCDK* genes. The phylogenetic tree on the left represents the *CDKs* from *P. mume* var. tortuosa and *A. thaliana*. The construction of the phylogenetic tree using the maximum likelihood method.

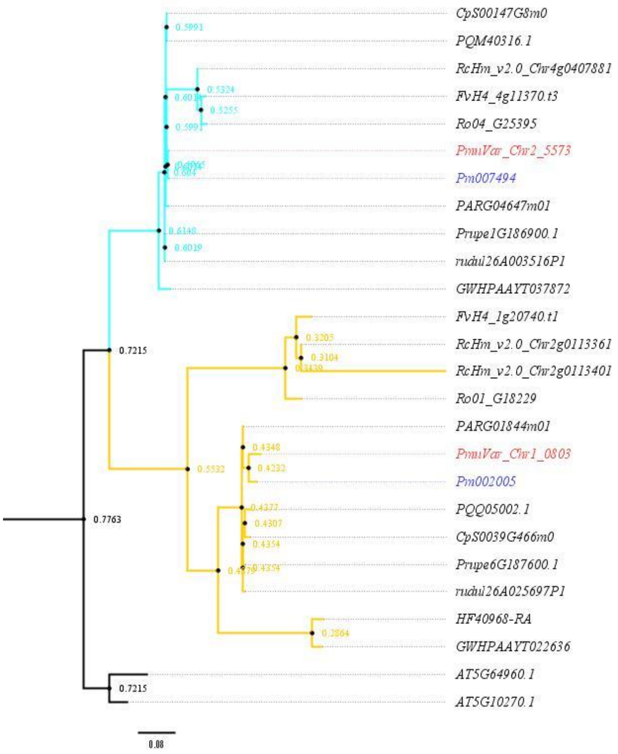

Fig. S14 A phylogenetic tree of *CDKC* genes from *P. mume* var. tortuosa and selected plants. The construction of the phylogenetic tree using the maximum likelihood method. Red and blue represent the genes of *P. mume* var. tortuosa and *P. mume*, respectively.

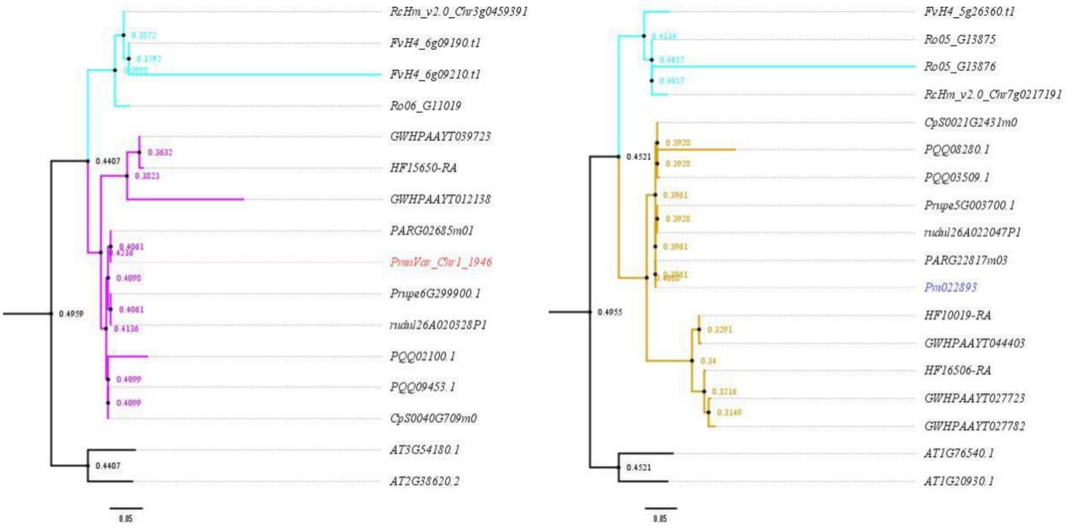

Fig. S15 The phylogenetic trees of *CDKB* genes (Left, *CDKB1* gene; Right, *CDKB2* gene) from *P. mume* var. tortuosa and selected plants. The construction of the phylogenetic tree using the maximum likelihood method. Red and blue represent the genes of *P. mume* var. tortuosa and *P. mume*, respectively.

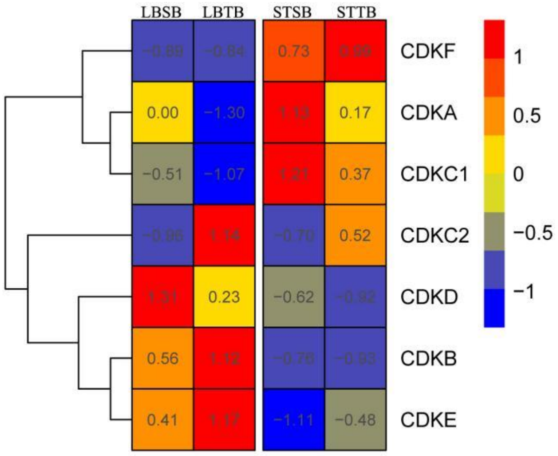

Fig. S16 Heat map for transcript abundance of the *PmCDK* genes. The amount of gene expression was standardized using the pheatmap function.

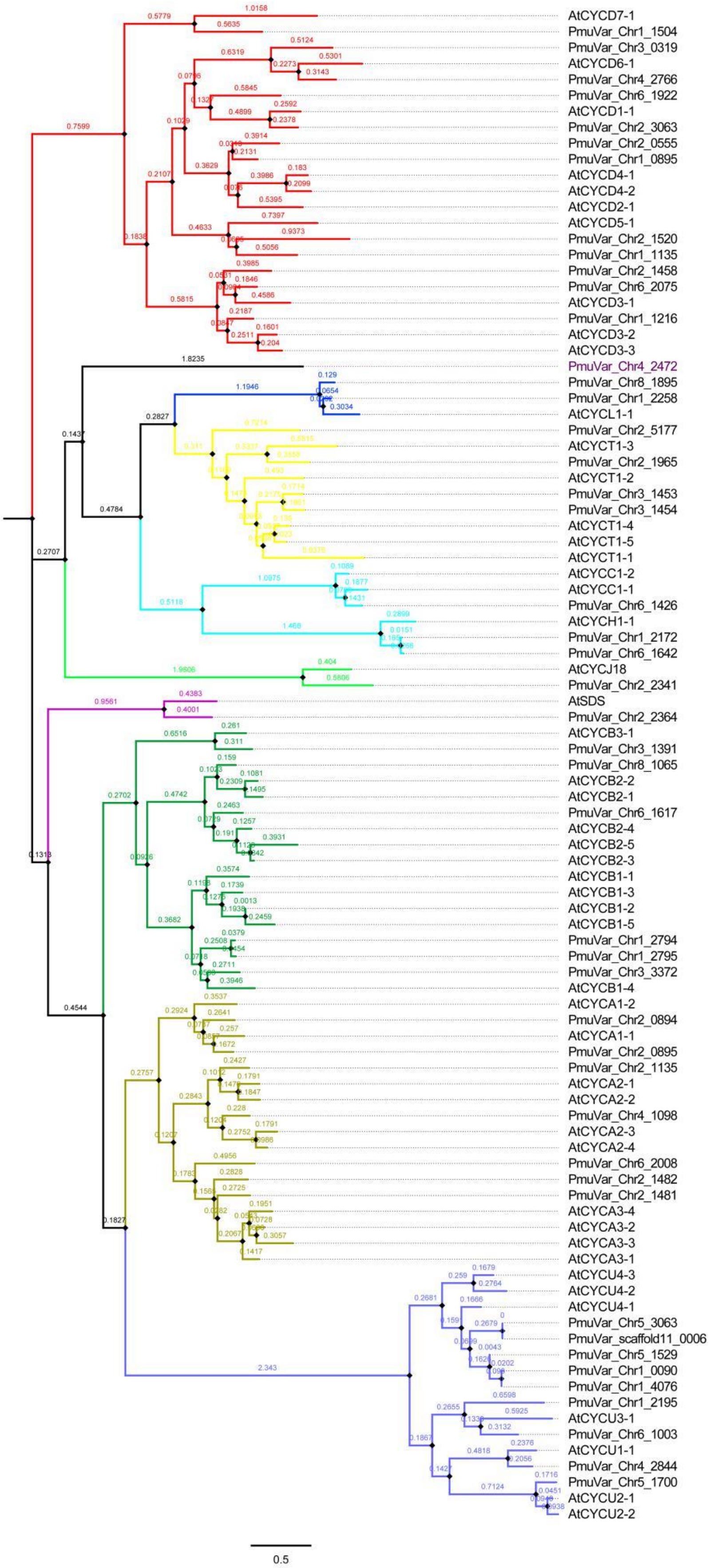

Fig. S17 A phylogenetic tree of cyclins from *P. mume* var. tortuosa and *A. thaliana*. The construction of

the phylogenetic tree using the maximum likelihood method. Red and blue represent the genes of *P. mume* var. tortuosa and *P. mume*, respectively.

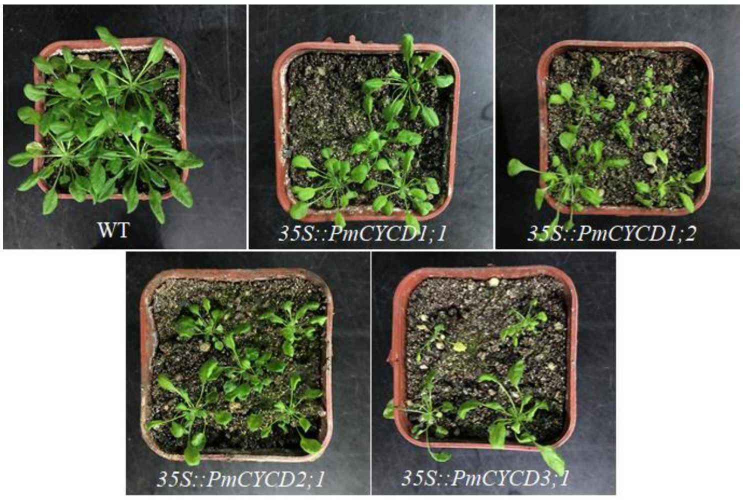

Fig. S18 Overexpressing *PmCYCDs* in *A. thaliana* with a rosette leaves curling phenotype

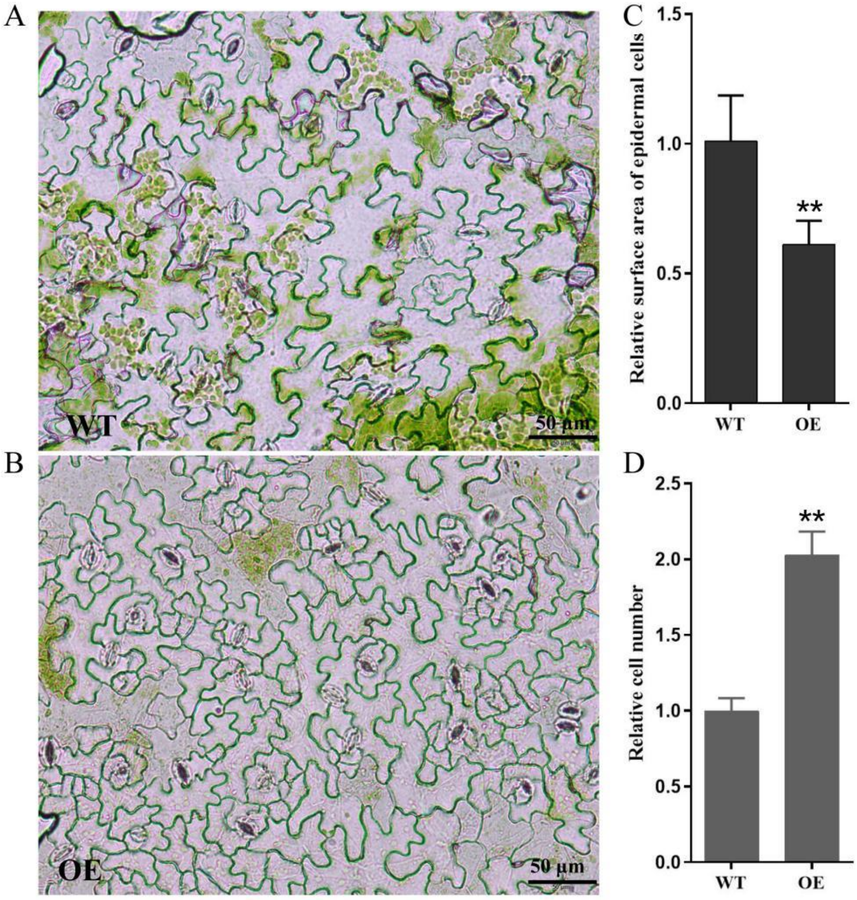

Fig. S19 The cellular level phenotype of the *PmCYCD1;2* overexpressing (OE) plants compared with wild type plant (WT). A and B. The epidermal cells of plants. C. Relative surface of epidermal cells. D. Relative cell number. The error bars represent +/- SD. **, difference was extremely significant ($P < 0.01$).

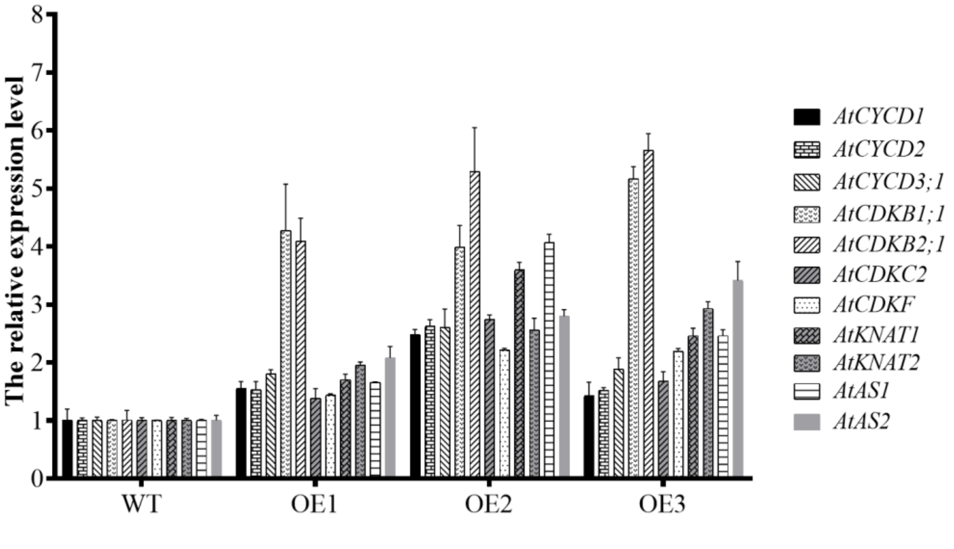

Fig. S20 Relative expression levels of tortuous-related genes. The error bars represent +/- SD.
